# Phosphorylation landscape of dengue virus proteins and their implications in protein-protein interactions

**DOI:** 10.1371/journal.pone.0345872

**Published:** 2026-05-12

**Authors:** Prakhar Agrawal, Rahul Yadav, Arumugam Madhumalar, Ganesan Senthil Kumar

**Affiliations:** 1 Integrative Structural Biology Laboratory, BRIC-National Institute of Immunology, New Delhi, India; 2 Multidisciplinary Centre for Advanced Research and Studies, Jamia Millia Islamia, New Delhi, India; Telethon Institute of Genetics and Medicine, ITALY

## Abstract

Phosphorylation is one of the most ubiquitous, reversible post-translational modifications through which cells communicate external signals from the membrane to the nucleus. However, viruses replicate in the host cells by hijacking the phosphorylation signaling to evade immune responses, as shown previously for Ebola and HIV. Here, we characterized the potential phosphorylation sites, the kinases involved, and their location in the structure of the Dengue virus proteins. We also show that the phosphosites in the four Dengue serotypes are evolutionarily conserved across other flaviviruses. Further, we show that the phosphorylation of dengue viral proteins is critical for mediating the interaction of these viral proteins with the host proteins, antibodies, and other viral proteins. In summary, we provide an extensive resource of phosphosites across the Dengue virus/flavivirus proteins that could be leveraged to understand the role of phosphorylation signaling in viral replication and develop novel drug therapies.

## Introduction

Viruses like the Dengue virus, upon infection, target cells to gain entry and use host cell machinery to replicate in the host. Infection of host cells by viruses is followed by virus internalization and viral genome release into the host cytoplasm. The Dengue virus genome is transported to the Endoplasmic Reticulum (ER), where it associates itself with the ER membrane protein complex for translation of the viral genome into polypeptides, as observed for the positive-stranded RNA viruses like Dengue [1,2]. Most importantly, the translation and maturation of viral proteins involve post-translational modifications such as N-linked glycosylation, glycosylphosphatidylinositol linkage, phosphorylation, and lipid raft association, among others. Most often, viruses effectively replicate in cells by hijacking the phosphorylation signaling, wherein the phosphorylated viral protein is involved in regulating viral genome replication, viral gene transcription, and recruitment of host proteins such as helicases [3–5]. Hence, it is important to inhibit viral replication by blocking the viral-dependent phosphorylation. Further, the addition of negatively charged phosphate groups alters the overall net charge on the proteins and, hence, leads to changes in the protein conformations, resulting in their increased or decreased functional stability [6].

Upon viral infection, several cellular signaling pathways (like the MAPK and Jak/STAT) get activated, leading to the phosphorylation of cellular and viral proteins [7]. For instance, West Nile Virus (WNV) infection of microglial cells is associated with increased cellular phosphorylation by the MAP kinases p38, ERK, and JNK. This phosphorylation and activation of p38, ERK, and JNK pathways induce chemokine and cytokine production in WNV-infected microglial cells [8]. Similarly, Epstein-Barr virus (EBV) latent membrane protein 1 (LMP1) activates the PI3K/Akt pathway, as LMP1 expression induces the phosphorylation of Akt; this signaling pathway is involved in the actin cytoskeleton reorganization of EBV-infected cells [9]. Hence, though there are many ways that viral infections can induce and/or inhibit cellular signaling pathways, the most common mechanism is the phosphorylation of cellular and viral proteins.

Most importantly, the phosphorylation is carried out by a plethora of kinases (> 550 kinases in the human genome), and hence, understanding the kinases involved in phosphorylating the viral proteins (substrates) and the biological functions carried out by these substrates is essential in understanding the viral pathogenesis and development of novel therapeutics [10]. The protein kinases phosphorylate the substrates by recognizing specific short linear motifs (SLiMs) [11]. Various bioinformatic tools have been developed to accurately predict the potential phosphorylation (PH) sites and the kinases that are involved in phosphorylating the substrates [12]. Among the different online tools, NetPhos3.1 predicts the phosphorylation sites in proteins and kinases involved in the eukaryotic system [13]. It considers 17 eukaryotic kinases such as, ATM (Ataxia telangiectasia mutated kinase), CKI (Casein kinase I), CKII (Casein kinase II), CaM-II (Calcium/calmodulin-dependent protein kinase II), DNAPK (DNA-dependent protein kinase), EGFR (Epidermal growth factor receptor kinase), GSK3 (Glycogen synthase kinase 3), INSR (Insulin receptor tyrosine kinase), PKA (Protein Kinase A), PKB (Protein Kinase B), PKC (Protein Kinase C), PKG (Protein Kinase G), RSK (Ribosomal S6 kinase), SRC (SRC proto-oncogene, non-receptor tyrosine kinase), cdc2 (cell division protein kinase 2), CDK5 (cyclin-dependent kinase 5), and p38MAPK (p38 mitogen-activated protein kinase).

Dengue virus (DENV) infection is the most prevalent arboviral disease, accounting for more than 390 million cases annually [14]. There are four DENV serotypes (DENV 1–4), and infection with any of the four serotypes produces a range of symptoms. This includes a mild flu-like illness or a life-threatening dengue hemorrhagic fever (DHF) and dengue shock syndrome (DSS). There is no clinically approved antiviral drug to treat DENV infection (with several potential candidates at different stages of clinical trials) except Dengvaxia, which is approved for limited use in a few countries. Besides, Dengvaxia is inefficient in treating all the DENV serotypes and has been shown to neutralize DENV-4 more effectively, while other serotypes are neutralized by cross-reactive antibodies [15,16]. Previous studies have shown that the phosphorylation of DENV NS1 affects the stability of the NS1 dimers, and the hyperphosphorylation of NS5 dissociates the NS3-NS5 complex to facilitate its transport to the nucleus [17,18]. Studies have also shown that the NS3-NS5 complex is essential for viral replication, and the phosphorylation of the S137 residue in NS3 increases its binding affinity to the NS5 proteins [19]. The phosphorylation of NS5 by PKG induces NS3-NS5 complex formation [19]. Most importantly, the current understanding of the role of phosphorylation in DENV is limited to several residues of NS1, NS3, and NS5 of DENV-2. Hence, it is crucial to identify all the potential phosphorylation sites of all the DENV viral proteins to understand how they function. Further, the presence of four serotypes with diverse sequences provides a distinct mechanism for viral replication for the four serotypes, and hence, it is also imperative to identify the similarities and differences in the phosphosites of DENV to understand the mechanism of their replication.

In this paper, we used bioinformatic tools to identify the potential phosphorylation sites of the various domains of Dengue viral proteins, using the DENV-2 serotype as the model system. First, we have identified the phosphorylation sites and their location in the structure, as well as the kinases that phosphorylate these sites in all three structural (glycoprotein E, C, and prM) and seven non-structural (NS1, NS2A, NS2B, NS3, NS4A, NS4B, and NS5) proteins. Then, to map the differences and similarities in the phosphosites of DENV serotypes, we identified the phosphosites across the other three DENV serotypes, namely DENV-1, DENV-3, and DENV-4. Next, we performed an evolutionary analysis to determine the phosphosites conserved across other flaviviruses. We show that many phosphosites are highly conserved across the multiple DENV serotypes and other flaviviruses, suggesting the importance of phosphorylation in mediating the interactions with host/viral proteins and antibodies. Lastly, we compared the interaction sites of the structural and non-structural proteins in complex with their known interacting partners (like antibodies, host proteins, and viral proteins) with the predicted phosphorylation sites to identify the potential role of phosphorylation in mediating the interaction with their interacting partners, which was further corroborated using molecular dynamics (MD) simulations. Thus, targeting the function of these highly conserved phosphosites across flaviviruses could open up avenues for developing novel therapeutics for treating flaviviruses’ infection.

## Computational methods

### Sequence analysis and phosphosite/kinase/secondary structure prediction

The dengue protein sequences were obtained from the NCBI database using dengue type 1, type 2, type 3, and type 4 as keywords to search (https://www.ncbi.nlm.nih.gov/protein/). The sequences thereby selected have the following GenBank accession IDs: JQ915078.1 (dengue type 1), KM204118.1 (dengue type 2; protein ID – AIU47320.1), AY496871.2 (dengue type 3), and MW945636.1 (dengue type 4). The protein sequences of individual proteins of DENV were then used as input for the NetPhos3.1 web server (https://services.healthtech.dtu.dk/services/NetPhos-3.1/) to predict the phosphorylation sites and the kinases that phosphorylate. In this server, the serine, threonine, and tyrosine phosphorylation states were predicted using ensemble neural networks. Further, this server determined the kinases that phosphorylate the predicted phosphosites. Using this server, the predictions can be made for only 17 kinases, namely, ATM, CKI, CKII, CaM-II, DNAPK, EGFR, GSK3, INSR, PKA, PKB, PKC, PKG, RSK, SRC, cdc2, CDK5, and p38 MAPK. The cutoff value for phosphosite prediction was kept at 0.5. Further, the residues for which no kinases were predicted were treated as outliers and removed from the data analysis. In the residues that predicted more than one kinase, the kinase with a higher prediction score was considered the kinase that phosphorylates that particular residue. Further, the structural and non-structural protein sequences from dengue serotypes were aligned with respect to dengue type 2 using BioEdit sequence analysis software [20]. To these aligned sequences, the predicted PHs were mapped. The kinases identified for dengue proteins and the prediction score were analyzed and plotted using Microsoft Excel (version 2021) and GraphPad Prism (version 10). The secondary structure for the location of the predicted PHs (random coil or loop, beta-turn, extended strand, and alpha helix) was deduced using the SOPMA Secondary Structure Prediction tool (https://npsa-prabi.ibcp.fr/cgi-bin/npsa_automat.pl?page=/NPSA/npsa_sopma.html). The percentage proportion of each secondary element for the PHs of individual proteins of the DENV was determined by making pie charts using GraphPad Prism (version 10).

### Mapping of the PHs on the crystal structure of dengue proteins

Previous structural studies have focused mainly on the Dengue virus serotype 2, which led to many crystal structures of dengue type 2 proteins with less or no significant structural information for the dengue virus types 1, 3, and 4. Hence, the predicted PH sites were mapped on the dengue type 2 proteins. The crystal structures of the respective dengue type 2 proteins (glycoprotein E: 7CTH, NS1: 6WER, NS3 protease: 2FOM, NS3 helicase: 8GZQ, and NS5: 8T12) were downloaded from the PDB database. Since PDB files have extra amino acids due to cloning exigencies, their sequence was aligned with the sequence taken from the NCBI database using the Clustal Omega tool (https://www.ebi.ac.uk/Tools/msa/clustalo/). Following alignment, the numbering of residues in the PDB file was adjusted to match the NCBI sequence numbering. The figures showing the phosphosites mapped on the structures were generated using PyMOL (https://PyMOL.org/2/).

### Identification of conserved PHs across serotypes and flaviviruses

To identify conserved PHs across serotypes, multiple sequence alignment was performed for each dengue protein using the Clustal Omega tool (https://www.ebi.ac.uk/Tools/msa/clustalo/). Conserved PHs determined were plotted for each protein as a Venn diagram using a Venn diagram web tool (https://bioinformatics.psb.ugent.be/webtools/Venn/). The identified conserved PHs were mapped on the protein structure using PyMOL. To further characterize the evolutionary conservation of PHs across flaviviruses, the sequence of DENV-2 protein was taken, and the Basic Local Alignment Search Tool (BLAST) was employed to identify similar sequences in flaviviruses, keeping the E threshold at 10 using the BLOSUM62 matrix. The NCBI sequence identifier was exported for each sequence identified for each flavivirus structural and non-structural protein. The identifier was then taken to individually export sequences from the NCBI database to a text document in fasta format. The sequence alignment was then performed using ClustalW and BioEdit sequence alignment software [20]. The alignment file thus generated was used to identify the presence of evolutionarily conserved PHs across flaviviruses, and the WebLogo (https://weblogo.berkeley.edu/logo.cgi) was used to generate an image file showing DENV protein regions with PHs conserved across flaviviruses. Furthermore, to identify conservation of phosphosites across the different circulating strains of Dengue serotypes, dengue serotype-specific sequences for E, NS1, NS3, and NS5 proteins were taken from the NCBI Virus database (https://www.ncbi.nlm.nih.gov/labs/virus/vssi/#/). The sequence was exported in Fasta format, and alignment was performed using ClustalW in BioEdit sequence alignment software. From the aligned file, the consensus sequence was exported, domain boundaries curated and aligned to the reference protein sequence (DENV-2) to identify predicted phosphosites conservation in the consensus sequence.

### Identification of residues of dengue proteins involved in host protein interactions and molecular docking of viral protein-antibody/host protein complexes

The residues involved in the dengue viral protein -viral/antibodies/host protein interactions were identified using the SPPIDER (Solvent accessibility-based Protein–Protein Interface iDEntification and Recognition) interface. SPPIDER determines the solvent accessibility of the amino acids and identifies the residues predicted to be involved in protein-protein interaction [21,22]. The results of SPPIDER were tabulated and visualized using PyMOL. Before performing HADDOCK (High Ambiguity Driven protein-protein DOCKing) docking for the phosphorylated and non-phosphorylated dengue proteins with their known interactors, PyTM plugin in PyMOL was used to convert non-phosphorylated Ser/Thr/Tyr residues to phosphorylated Ser/Thr/Tyr residues and subsequently, modified PDB files were generated using PyMOL. HADDOCK docking (https://rascar.science.uu.nl/haddock2.4/) was then performed using phosphorylated dengue protein as a receptor and its known interactor as a ligand. The modified PDB files were used as inputs. The following residues were defined as interacting residues for the HADDOCK docking: glycoprotein E (E147, E148, H149, V309, K310, R323, Q325, E360, D362, and P364), mAb ScFv EDE1 C10 (N31, Y49, D50, T52, S53, R54, and S60), NS3 protease (E19, D20, G21, A22, Y23, R24, I25, K26, Q27, K28, L31, G32, Y33, S34, Q35, I36, V40, F46, A56, V57, L58, M59, H60, K61, Q96, L98, L100, P102, G103, N105, P106, R107, A108, V109, Q110, V140, D141, K142, G144, and V146), NS2B (L47, A49, D50, L51, E52, L53, E54, R55, A56, A57, D58, V59, R60, W61, E62, A65, E66, G69, S70, S71, P72, I73, S75, K87, N88, E90, E92, Q93, and L95), NS3 helicase (K185, R187, N188, L189, I191, T303, G306, M307, E309, P319, P320, D324, F326, D335, E337, R338, D339, T436, D437, I443, A445, G446, P449, D485, I505, F510, E511, P512, and R514), NS5 for NS3 helicase interaction (T224, M233, R236, N240, T243, M244, A312, G322, K585, G586, W746, S747, L748, K749, L873, I874, G875, and N876), NS5 for hSTAT2 interaction (R47, G107, P108, G109, K312, Q313, T314, G315, S316, A317, S318, S319, M320, V321, G323, R326, L327, K330, I334, I335, P336, M337, Q340, A342, K461, L462, F465, D690, R698, N730, D732, W746, S747, L748, T751, Q845, W846, G848, S849, L850, L853, T854, S855, T858, L873, and E877), and hSTAT2 (H24, D52, D53, S54, T57, M58, F60, F61, L64, N68, H85, R92, Q95, P96, S98, Q99, P101, Q165, D168, Q169, D171, V172, F175, R176, K178, I179, Q180, K182, G183, T185, S187, L188, D189, P190, H191, K194, K197, I198, E201, T202, N204, E205, D207, and D290). The HADDOCK server automatically defined the active and passive residues from these interacting residues based on solvent accessibility. The HADDOCK parameters for biomolecular docking were kept at their default values, followed by energy minimization and HADDOCK docking. Once the run was complete, clusters with the lowest docking scores were selected, and the results were analyzed using PyMOL and Chimera. The electrostatic potential of the phosphorylated and non-phosphorylated dengue proteins was calculated using the APBS web server (https://server.poissonboltzmann.org) using the PARSE forcefield with default parameters [23]. dG of binding was calculated using Prime module of Schrödinger software using default parameters (Schrödinger Release 2024−1: Prime, Schrödinger, LLC, New York, NY, 2024). The output generated (PQR file, grid file, and potential map) was imported into PyMOL to map the electrostatic potential on the structures.

### Molecular dynamics simulations

To prepare the phosphorylated and non-phosphorylated glycoprotein E-mAb ScFv EDE1 C10 and NS5-hSTAT2 complexes for MD simulations, the HADDOCK models were imported into Maestro module (Schrödinger Release 2024−1: Maestro, Schrödinger, LLC, New York, NY, 2024). The protein preparation wizard was used to add acetyl and methyl groups at the N and C-terminus of the protein. The bond orders were assigned, and structures were minimized using the OPLS2004 force field [24]. Then, the system builder tool embedded in the Desmond module was utilized to solvate the proteins in a simulation box (cubic with 10 Å length) containing sodium or chloride ions to neutralize the net charge on the protein. The simulation box containing the protein was then energy minimized via the minimization tool of the Desmond module (steepest decent model with 50000 steps). The MD simulations were carried out using the Desmond simulation package of Schrödinger LLC [25]. The NPT ensemble with a temperature of 300 K and a pressure of 1 bar was applied in all runs. The simulation length was 100 ns with a relaxation time of 1 ps for the phosphorylated and non-phosphorylated complexes. The OPLS force field parameters were used in all the simulations. The long-range electrostatic interactions were calculated using the particle mesh Ewald method. The cutoff radius for the coulombic interactions was 9 Å. The water molecules were explicitly described using the TIP3P model. The Martyna-Tuckerman-Klein chain coupling scheme with a coupling constant of 2.0 ps was used for pressure control, and the Nose-Hoover chain coupling scheme for temperature control. Nonbonded forces were calculated using an r-REPSA integrator, where the short-range forces were updated every step, and the long-range forces were updated every three steps. The trajectories were saved at 5 ps intervals for the data analysis. The advanced trajectory analysis tool implemented in the Desmond MD package analyzed the protein-protein behavior and interactions. The stability of MD simulations was monitored by looking at the RMSD and RMSF plots of the protein with respect to time.

## Results

### Domain construction of DENV Serotype 2

**Fig 1:**
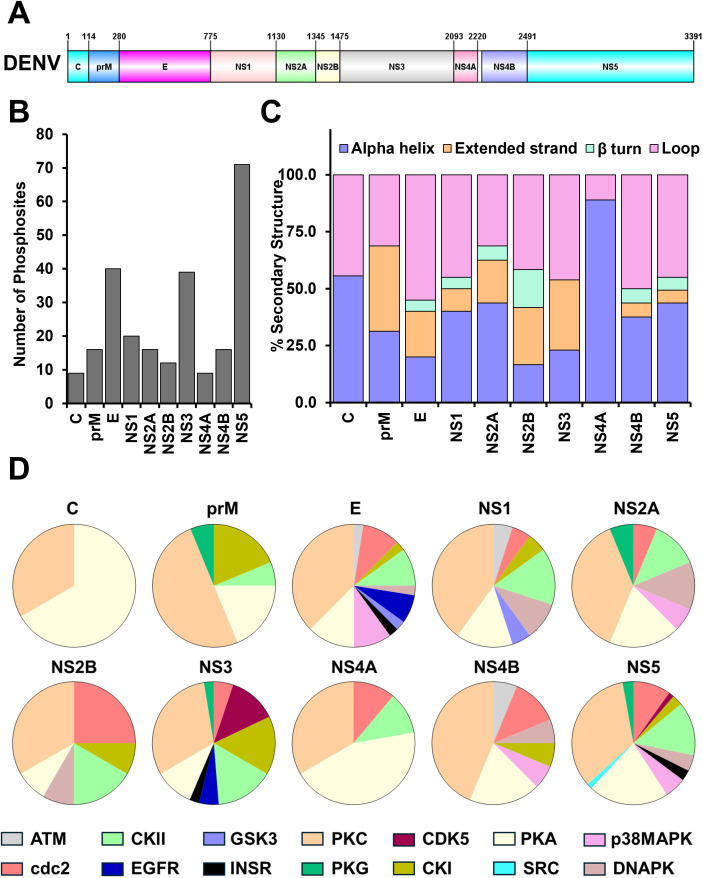
Phosphosite and kinase identification of structural and non-structural proteins of Dengue virus type 2. **(A)** Domain organization of dengue virus type 2. Also given are the residue numbers corresponding to each of the structural (C, prM, and glycoprotein E) and non-structural protein (NS1, NS2A, NS2B, NS3, NS4A, NS4B, and NS5). **(B)** Plot showing the number of predicted phosphosites as a function of Dengue viral proteins using a cutoff value of 0.5. Note that the non-structural proteins NS5 and NS3, and the structural glycoprotein E have the maximum number of predicted phosphosites. **(C)** Bar diagram showing the secondary structural elements where the predicted phosphosites are located. The alpha helix is shown in blue, the β-turn in cyan, extended strands in orange, and loops in pink. Note that most of the phosphosites are localized to the loop regions, except for heavily α-helical proteins like the C, prM, and NS4A. **(D)** Graphical representation of the kinases that phosphorylate the various proteins of Dengue virus. All the 14 kinases (except CaM-II, RSK, and PKB, where there is no predicted PH) are differentially colored (ATM-light grey, CKII-light green, GSK3-light blue, PKC-light orange, CDK5-raspberry, PKA-light yellow, p38MAPK-light pink, cdc2-dark pink, EGFR-blue, INSR-black, PKG-green, CKI-yellowish green, SRC-cyan, and DNAPK-light chocolate). Note that most of the sites are phosphorylated by Protein kinase A (PKA) and Protein kinase C (PKC).

Dengue virus (DENV) is an enveloped, positive-sense, single-stranded RNA virus (~11 kb) in the genus *Flavivirus* of the family *Flaviviridae* that includes four antigenically distinct serotypes. Infection of target cells with DENV serotype 2 (New Guinea C strain; henceforth referred to as DENV-2; GenBank: KM204118.1; protein ID – AIU47320.1) results in the production of three structural proteins, namely, capsid (C) protein (13.2 kDa; residues 1–114; harboring an N terminal intrinsic disordered region, an intermediate flexible fold region, and a conserved alpha-helical fold), pre-membrane (prM) protein [18.8 kDa; residues 115–280; harbors a membrane binding region (131–166)], and envelope glycoprotein E (52.28 kDa; residues 281–775; contains three ectodomains EDI, EDII, and EDIII along with a STEM region and a transmembrane helix at the C-terminus) that are required for virion formation, and seven non-structural proteins NS1 (39.9 kDa; residues 776–1130), NS2A (23.2 kDa; residues 1131–1345; comprised of five transmembrane domain, which spans the membrane of endoplasmic reticulum), NS2B (14 kDa; residues 1346–1475), NS3 [69.3 kDa; residues 1476–2093; composed of a chymotrypsin like serine protease domain followed by interdomain linker (IDR region) and an RNA helicase domain]., NS4A (14 kDa; residues 2094–2220), NS4B (26.76 kDa; residues 2244–2491), and NS5 [103.2 kDa; residues 2492–3391; composed of an N terminal methyltransferase domain and a C-terminal RNA dependent RNA polymerase (RdRp) connected by an intrinsically disordered region] that play essential roles in polyprotein processing and viral RNA replication (**Fig 1A**).

### *In silico* prediction of potential phosphorylation sites in the viral proteins of DENV-2

Phosphorylation of viral proteins plays an important role in their activity, stability, and interaction with other cellular proteins (that may or may not be phosphorylated) upon infection [26]. Previous studies have demonstrated that the phosphorylation of viral proteins in the SARS-CoV-2 virus is critical for its assembly and function [27,28]. Hence, it is important to characterize the phosphorylation states of viral proteins, which are poorly understood for various viral infections. To identify the phosphorylation sites in the viral proteins of the Dengue virus, we performed detailed bioinformatics analysis using the protein sequences of individual domains of the DENV-2 viral genome using NetPhos3.1 [13].

The sequences of the individual proteins of DENV-2 were analyzed to identify the potential phosphorylation sites. The number of phosphosites predicted for structural proteins C, prM, and glycoprotein E are 9, 16, and 40, respectively (**Fig 1B**, **Table 1, S1 Fig**). In contrast, the non-structural proteins NS1, NS2A, NS2B, NS3, NS4A, NS4B, and NS5 are predicted to harbor 20, 16, 12, 39, 9, 16, and 71 phosphosites, respectively (**Fig 1B**, **Table 1**, **S1** **and** **S2 Figs**.). Further, the percentage of phosphosites (with respect to the total number of residues) predicted across the various structural and non-structural proteins ranged from 5.68% to 9.64%, with only three proteins (prM, E, and NS2B) showing more than 8% predicted phosphosites (**Table 1**). This indicates that the phosphosites are located uniformly across the various domains of the DENV-2 genome. Most importantly, the majority of the predicted phosphosites (>40%, except the predominantly α-helical transmembrane proteins prM, NS2A, and NS4A) are localized in the loop/irregular structure regions, which correlates well with the observance of phosphosites at the loop/irregular structured regions in other human proteins (**Fig 1C**, **S1** **and** **S2 Figs**.) [29]. The glycoprotein E (55% loop, 20% α-helix, 20% extended strand, 5% β-turn), non-structural protein NS3 (46.15% loop, 23.08% α-helix, and 30.8% extended strand), and non-structural protein NS5 (45.1% loop, 43.7% α-helix, 5.6% extended strand, 5.6% β-turn), the three largest proteins of the DENV-2 genome, showed higher propensities for phosphorylation compared to other proteins of DENV-2 (**Fig 1C**, **S1** **and** **S2 Figs**.).

**Table 1:** Distribution of phosphosites across the various viral proteins of DENV2.

DENV Protein	No. of Amino Acids (y)	No. of Potential Phosphorylation Sites (x)	(x)/(y) (%)	Residues phosphorylated
**C Protein**	114	9	7.89	T11, S24, T25, T300, S34, T580, T 71, S75, T101
**prM Protein**	166	16	9.64	T4, S15, S22, T50, S70, T73, T76, T79, S92, T105, S112, S113, T125, T134, T146, T159
**E Protein**	495	40	8.08	S7, T32, T33, T55, T76, S81, T115, T120, Y137, T142, S145, T155, T165, S169, T171, Y178, S186, T236, T239, S255, T265, S273, T280, S298, T303, S331, T359, S363, Y377, S396, S397, T404, T405, S424, Y444, S472, T473, S474, S476, S478.
**NS1**	352	20	5.68	S7, T27, S38, S44, S80, S103, T117, T126, S152, S204, T209, S216, T230, S239, S252, T262, S297, T300, S304, S315.
**NS2A**	218	16	7.34	S9, T29, T86, T97, S112, S114, T119, T124, S168, S178, T183, S185, T209, T210, S212, T214.
**NS2B**	130	12	9.23	S19, S20, T39, T45, S48, S68, S71, T77, S83, S85, S107, S113.
**NS3**	618	39	6.31	S9, Y23, S78, T122, S131, T134, S137, T156, S158, S163, T168, S171, T189, T200, T218, T224, T244, T252, T266, S271, S293, S301, T302, T317, S345, T352, T358, S386, Y394, T407, T408, T435, S452, S453, Y472, T500, S507, T583, S602.
**NS4A**	127	9	7.09	S1, T15, T18, S46, T54, T63, S74, T82, S92.
**NS4B**	248	16	6.45	T19, T20, S25, S36, T45, T49, S55, T66, S100, T130, T195, T198, T215, S228, S238, T246
**NS5**	900	71	7.89	T8, S15, S31, S56, S59, T72, S88, Y89, T104, S117, T118, S128, T135, S150, T176, S189, S214, S231, T244, T251, S260, S269, T302, Y309, T311, T314, S316, S318, S319, T329, T347, T363, T370, T377, T390, T400, S405, T414, S471, S503, S505, S523, T540, T554, T572, T584, S601, T606, T613, S631, T636, S656, S661, S676, T704, S741, S747, S785, T790, T793, S796, T806, T811, T828, S832, S849, T854, T858, Y879, T880, S885

### Identification of host kinases involved in phosphorylating DENV-2 viral proteins

Viruses hijack the phosphorylation signaling, which is mediated by the kinases and phosphatases, to survive inside the host cell upon infection [7]. Kinases play a variety of roles in the viral lifecycle that include viral entry by regulating the phosphorylation of receptors, viral maturation by phosphorylating the capsid protein leading to its disassembly, and viral replication by modulating the host transcriptional and translational machinery [7,30–32]. For instance, previous studies have shown that the host kinases like protein kinase C (PKC), mitogen-activated protein kinase (MAPK), phosphoinositide 3-kinase (PI3K), and focal adhesion kinase (FAK) are crucial for the influenza virus entry and replication [33–36]. Similarly, in SARS-CoV-2, several host protein kinase families, like the serine-arginine protein kinase (SRPK), glycogen synthase kinase-3 (GSK-3), and casein kinase (CK1), phosphorylate the Nucleocapsid (N) protein, and their inhibition leads to impaired viral growth [36]. The Ser/Thr kinases protein kinase A (PKA) and protein kinase C (PKC) also play crucial roles in signal transduction, wherein PKA in host cells regulates metabolic cycles by phosphorylating the host transcription factors, resulting in their nuclear translocation, and phosphorylation by PKC leads to the activation of transcription along with the desensitization of the receptor proteins [37,38]. The casein kinases CKI and CKII phosphorylate various transcription factors to regulate transcription [39]. Thus, these kinases play a critical role in transcription, signal transduction, membrane structure modulation, and receptor desensitization, which are essential for virus replication upon infection. Further, the use of various kinase inhibitors or the knockdown of kinases like PKG, AKT, CDK1, and PRK2 has been shown to inhibit the growth of the viruses [40–45]. Hence, it is important to identify the kinases that regulate the phosphorylation state of the dengue viral proteins and their function.

To identify the kinases that potentially phosphorylate the DENV-2 proteins, we used NetPhos3.1. Our results show that 14 kinases (with a cutoff score of more than 0.5) are involved in the phosphorylation of DENV-2 proteins (**Fig 1D**, **S1** **and** **S2 Figs**.). Most importantly, protein kinases PKA and PKC were found to phosphorylate all the structural and non-structural proteins of DENV-2, with PKC phosphorylating 90 residues across the DENV-2 proteins (**Fig 1D**, **S1** **and** **S2 Figs**.). In addition, PKC has been predicted to phosphorylate more than 30% of the predicted phosphosites in all three structural and seven non-structural proteins (**Fig 1D**) except for the NS3 protein (27% with 12 phosphosites). In the case of NS2B and NS4B, PKC phosphorylates more than 55% of the predicted phosphosites. Interestingly, the two most conserved and most significant proteins of DENV-2, glycoprotein E and NS5, are phosphorylated by PKC to a larger extent, wherein glycoprotein E is phosphorylated at 15 sites (34%) and NS5 at 24 sites (43%). The other kinases that are predicted to phosphorylate multiple sites (> 10% of predicted phosphosites) across the DENV-2 proteins are CKII, cdc2, CDK5, CKI, DNAPK, PKA, and PKG (**Fig 1D**, **Table 1**, **S1** **and** **S2 Figs**.). While CKII phosphorylates all DENV-2 proteins except the C and NS4B proteins, the Cdc2 phosphorylates all but the C and prM proteins. CKI phosphorylates all except the C, NS2A, and NS4A proteins. On the other hand, DNAPK phosphorylates only the E, NS1, NS2A, NS2B, NS4B, and NS5 proteins among all the DENV-2 proteins. PKG is predicted to phosphorylate the prM, NS2A, NS3, and NS5 proteins (**Fig 1D**, **Table 1**, **S1** **and** **S2 Figs**.). Hence, our results suggest that multiple kinases are involved in the phosphorylation of DENV-2 proteins and could play critical roles in their biological function and survival in the host cells depending on the location of the virus and the stage of their life cycle.

### Conservation of phosphosites and kinases across the Dengue serotypes

**Fig 2:**
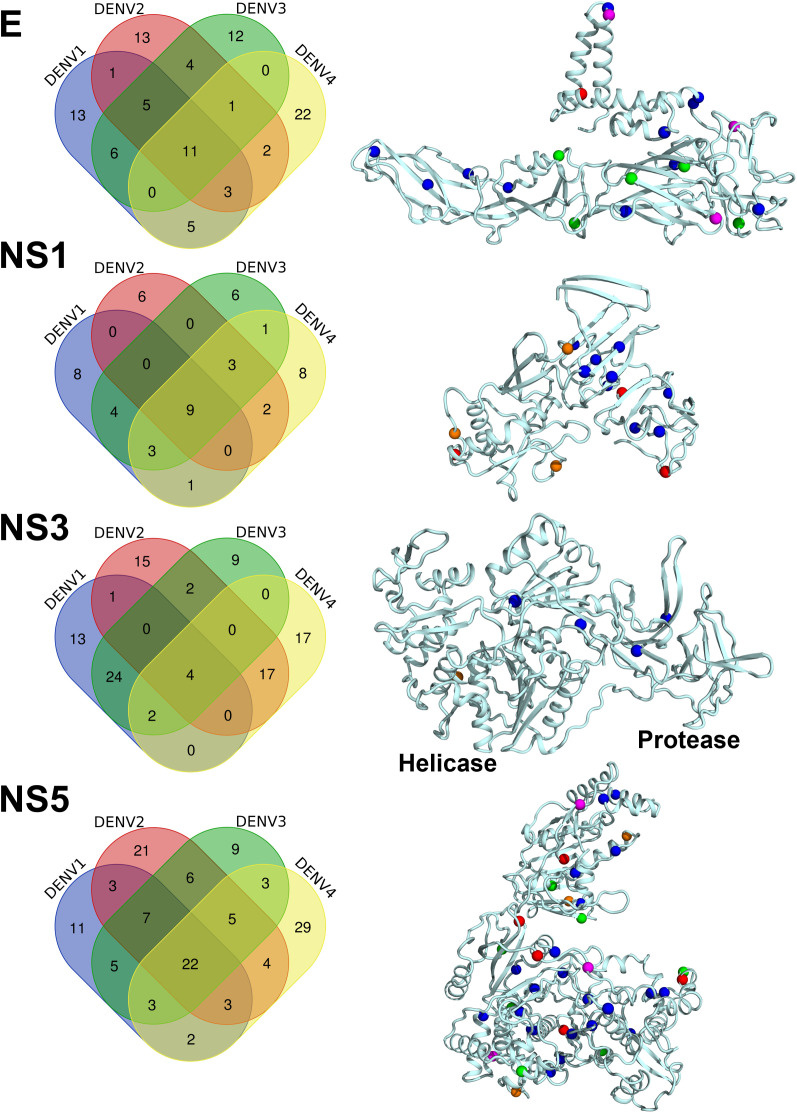
Comparison of PH sites of DENV-2 with other DENV serotypes. ***Left panel***- Venn diagram showing the predicted phosphosites for the glycoprotein E, NS1, NS3, and NS5 in Dengue virus serotypes 1, 2, 3, and 4. Note the number of overlapping PH sites across multiple DENV serotypes. ***Right panel*** – Structural mapping of PH sites that are identical in four serotypes (DENV 1, 2, 3, and 4; blue spheres), three serotypes (DENV 1, 2, and 3; green spheres), three serotypes (DENV 1, 2, and 4; magenta spheres), three serotypes (DENV 1, 3, and 4; orange spheres) and three serotypes (DENV 2, 3, and 4; red spheres) onto the crystal structures of glycoprotein E (PDB ID: 7CTH), NS1 (PDB ID: 6WER), NS3 (PDB ID: 5YW1), and NS5 (PDB ID: 8T12). Note that the conserved phosphosites are located throughout the Dengue proteins.

The major challenge in the design of vaccines/drugs for the treatment of dengue virus infection is the prevalence of four major serotypes, namely DENV-1, DENV-2, DENV-3, and DENV-4. Most importantly, the sequence of these serotypes is significantly different, with the non-structural protein 5 (NS5) and the DIII domain of glycoprotein E being the most conserved. Given the little sequence conservation across dengue serotypes, we sought to identify the conserved phosphosites across the serotypes for structural and non-structural dengue proteins. Our results show that the number of phosphosites that are conserved across the four serotypes includes four in prM, two in C, 11 in glycoprotein E, nine in NS1, one in NS2A, four in NS3, four in NS4B, and 22 in NS5 (**Fig 2**, **S3**, **S4**, **S5**, **S6**, **S7**, **S8** **and** **S9A Figs**.). Further, we found that no phosphosites are conserved in NS2B and NS4A (**S9A Fig**). Notably, upon a comparison of residues phosphorylated amongst serotypes (**Fig 2**, **S1**–**S3 Table**), phosphosite locations were observed to be different for dengue proteins in all serotypes in some residues, which could be due to the differences in the sequences across the DENV serotypes. However, the total number of phosphosites predicted for each serotype remained fairly constant at 7.07–7.36% phosphosites w.r.t total number of residues (248 out of 3368 residues in DENV-2, 242 out of 3373 residues in DENV-1, 240 out of 3391 and 3364 residues in DENV-3 and DENV-4, respectively; **Table 1**, **S1**, **S2****, and** **S3 Table**). In addition, 47 phosphosites present in various Dengue virus proteins are conserved in at least three serotypes, with 18 phosphosites located in the NS5. Further, most of these phosphosites were localized in the loop regions of the structural and non-structural proteins, much like the DENV-2 proteins (**S1**, **S2**, **S3**, **S4**, **S5**, **S6**, **S7****, and** **S8 Figs**.). Mapping these conserved phosphosites on the dengue proteome shows that these regions correspond to protein-protein interaction sites. For example, the conserved phosphosite S255, across the DENV serotypes, is located at domain II, which is known to interact with domain III, leading to dimerization and trimerization [46]. Also, DENV-2-specific phosphosite S7 in domain I is involved in the dimerization. Hence, two of the four Ser/Thr/Tyr residues involved in the dimerization of glycoprotein E are predicted to be phosphorylated, suggesting a crucial role of phosphorylation in the dimerization of glycoprotein, leading to virion assembly. It is highly plausible that the remaining conserved phosphosites (T32, T76, T115, T165, T239, S298, S396, S397, S424, T473), along with the aforementioned phosphosites, could be involved in binding with prM and cell surface receptors/antibodies (**Fig 2**, **Table 1**). Importantly, the four conserved phosphosites (T50, T79, S92, S112) in prM protein (**S9A Fig****., Table 1**) might also explain the stringent control of E protein association with prM that determines the viral maturation. In the case of NS1, while the residues T27, S204, S216, T230, S239, S252, S297, T300, and S315 were found to be conserved across the serotypes; the residues S114, T140, and S185 (in DENV 1, 3, and 4) and T117, T262, and S304 (DENV 2, 3, and 4) were found to be conserved in at least three serotypes. Among these, residues T27 and T230, which are conserved across all DENV serotypes, are known to play vital roles in NS1 dimerization and subsequent multimeric structure formation, in addition to their interaction with the antibodies. Similarly, in the case of NS3, the residue Y23 (conserved across serotypes) is known to interact with NS2B and T302, and T435 is involved in the interaction with NS5. Only in NS5, the residues that are found conserved across the serotypes (T8, S31, S56, T104, S150, S214, T329, T347, T363, S471, S503, S505, T540, T572, S601, T606, T613, S741, S747, T793, S796, and S849), except S747 and S849, were found to be farther away (> 6 Å) from the interacting sites of NS3/STAT2. Most likely, in NS5, the conserved phosphosites, despite not being involved in the direct interaction, could either play an indirect allosteric effect in mediating the interactions with NS3 and/or STAT2 or could be part of yet uncharacterized protein-protein interaction networks (**Fig 2**). The role of conserved phosphosites in C protein (T25 and S34), NS2A (T97), and NS4B (T130, T215, S228, and S238) are not very clear but are most likely important for various protein-protein interactions. Among all the domains, only NS2B and NS4A do not contain any conserved phosphosites across the four DENV serotypes.

As seen in the case of DENV-2, the top four kinases common across all proteins were PKC, PKA, CKI, and CKII (**Fig 1 and** **S1**, **S2**, **S3**, **S4**, **S5**, **S6**, **S7****, and** **S8 Figs**). They were found phosphorylating all DENV-1 proteins, with exceptions in the case of C protein (CKI and CKII absent), E protein (CKII absent), NS2B (PKA, CKI absent), and NS4A (CKI absent). Further, kinases such as RSK and SRC were found to be phosphorylating exclusively the C protein, NS3, and NS5, respectively. Protein kinase PKC and PKA phosphorylate all the proteins of DENV-3, whereas CKII was found phosphorylating all except C, prM, NS2A, and NS4A. Similarly, except for NS4B, all other proteins were predicted to be phosphorylated by cdc2. CKI and CKII kinases were found phosphorylating glycoprotein E, NS1, NS2B, NS3, NS4B, and NS5 (**S1**, **S2**, **S3**, **S4**, **S5**, **S6**, **S7****, and** **S8 Figs**). In the case of DENV-4 proteins, PKA and PKC were found to phosphorylate all the proteins, whereas other kinases like DNAPK, cdc2, CKII, and CDK5 were phosphorylating one or the other proteins in DENV-4 (**S1**, **S2**, **S3**, **S4**, **S5**, **S6**, **S7****, and** **S8 Figs**.). In summary, our results show that the kinases PKA and PKC play a critical role in the phosphorylation of the majority of the structural and non-structural proteins across the DENV serotypes, suggesting that the cellular processes mediated by these kinases could be hijacked by the Dengue virus for its propagation.

### Evolutionary analysis to identify the conserved phosphosites across the flaviviruses

**Fig 3:**
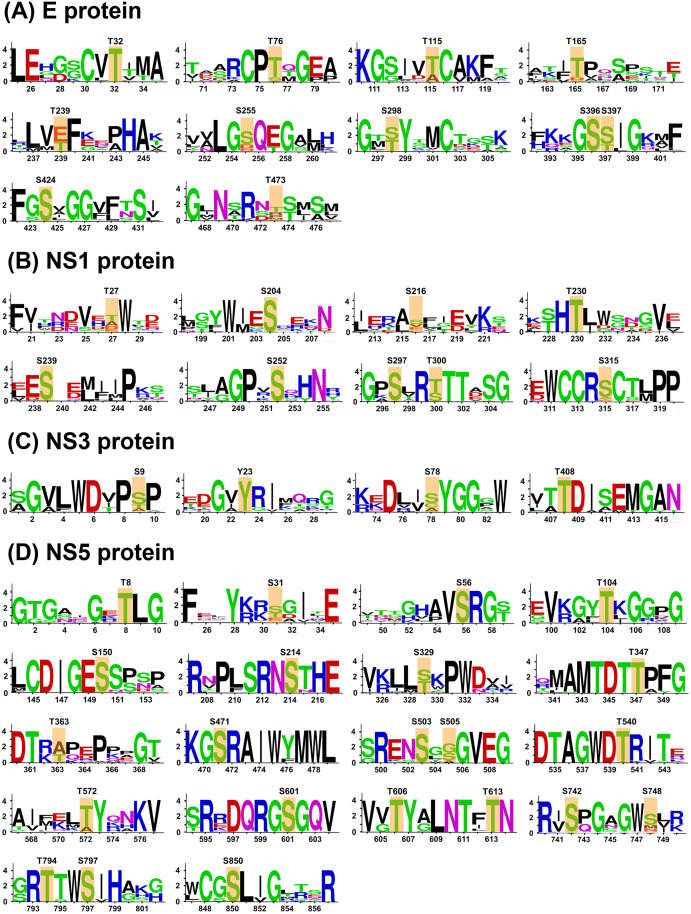
Conservation of PH sites across the flaviviruses. Web logo showing the phosphorylation cluster across the sequences in the glycoprotein E, NS1, NS3, and NS5 proteins of flaviviruses. The sequence motifs near the conserved phosphosites in DENV serotypes are shown. The conserved phosphosites from the DENV serotypes are highlighted in an orange box, and the bits show the degree of conservation across flaviviruses (bit value of 4 represents a highly conserved residue, whereas a value of 1 shows the least conservation at that position). Note that the majority of phosphosites that are conserved across the DENV serotypes are conserved across the flaviviruses.

Previous studies have shown that there are more than 70 viruses (like Dengue, West Nile, Japanese encephalitis, and Yellow Fever, among others) that belong to the genus *Flavivirus* of the family *Flaviviridae* [47]. The evolutionary correlation among the flaviviruses using phylogenetic analysis has been well-established [48]. Interestingly, the various domains (both the structural and non-structural proteins) are highly conserved across the flaviviruses [49]. Hence, we performed a sequence analysis of the flavivirus family (that includes multiple sequences from different circulating DENV serotypes; 28 unique DENV sequences were used, the accession numbers of the flavivirus sequences used are provided in **S4**, **S5**, **S6**, **S7**, **S8**, **S9**, **S10****, and** **S11 Table**) to test whether the phosphosites that are conserved across the DENV serotypes are also conserved in other flaviviruses. Here, we have used the sequence of DENV-2 proteins, and the sequences of the other flaviviruses were identified using BLAST analysis. Subsequently, multiple sequence alignment was performed to identify the conserved phosphorylation motifs (**Fig 3A-3D**, **and** **S9A**, **S9B**, **S10**, **S11****, and** **S12 Figs**). For instance, in E protein, three phosphosites (T32, S396, and S424) and eight phosphosites (T76, T115, T165, T239, S255, S298, S397, and T473) were absolutely conserved and fairly conserved, respectively, in other flaviviruses like WNV, Zika, JEV, and Yellow Fever virus, among others (**Fig 3A and** **S10 Fig**.). In prM, residues T50, T79, S92, and S112 were found conserved to varying degrees across flaviviruses (**S9****B and** **S10, Fig**.). In C protein, 25T and 34S are highly and partially conserved, respectively (**S9****B and** **S10 Fig**.). Similarly, S204, T230, S239, S252, S297 were found highly conserved whereas T27, S216, S315 were partially conserved in case of NS1 (**Fig 3B and** **S10 Fig**.). In NS3, Y23 and T408 were found to be highly conserved (**Fig 3C**, **S11 Fig**), while S9 and S78 are partially conserved. In the case of NS5, 16 out of the 22 DENV conserved phosphosites, namely, T8, S56, T104, S150, S214, S347, S471, S503, T540, S601, T606, T613, S742, T794, S797, and S850 were completely conserved in flaviviruses, suggesting the importance of NS5 and its phosphorylation in viral replication (**Fig 3D and** **S12 Fig**.). Lastly, the NS2A and NS4B protein has less conservation in phosphosites across the flaviviruses (**S9**, **S11** **and** **S12 Figs**). Overall, 27 out of the 57 DENV conserved phosphosites are conserved across other flaviviruses, suggesting a conserved phosphorylation mechanism for the flavivirus infection and propagation. It is also important to note that the sequences flanking the phosphosites are also highly conserved across the flaviviruses, suggesting that these regions could be important for critical protein-protein interactions that determine the outcome of the viral infection. Our results show that the majority of the phosphosites are conserved across the flaviviruses with the exception of several sites in other flaviviruses, that could be due to the evolution of mutants or different strains that were considered in this study. This high degree of conservation across the flaviviruses shows that they are evolutionarily co-evolved, and targeting these highly conserved phosphosites (and their subsequent function) could lead to the development of novel therapeutics for the universal treatment of flavivirus infection.

### Effect of phosphorylation on the known host-pathogen/antigen-antibody interactions of DENV-2 proteins

**Fig 4:**
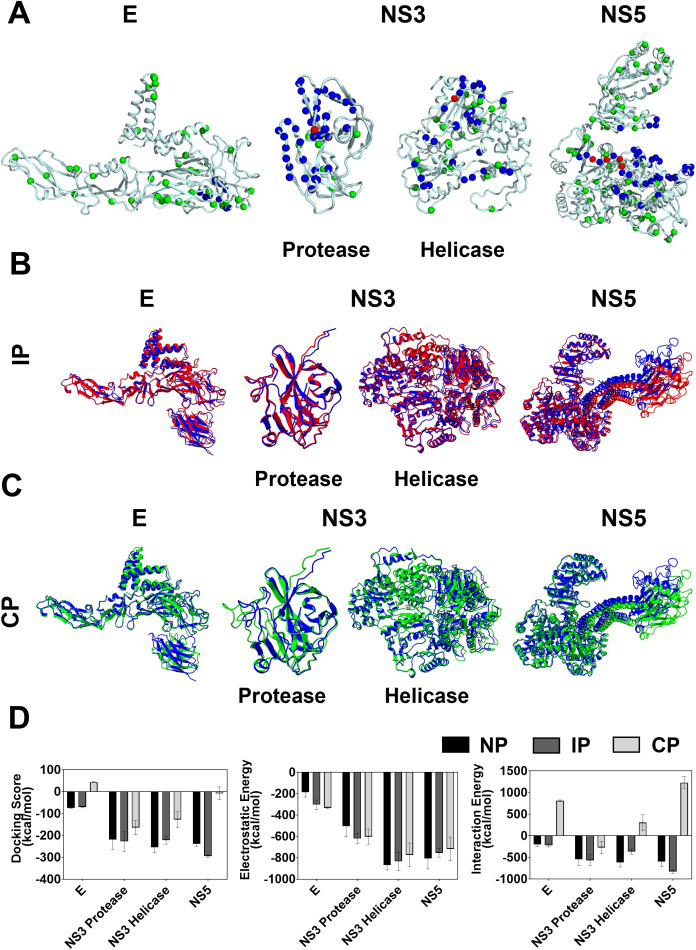
Effect of phosphorylation on the known host-pathogen and antigen-antibody interactions. **(A)** Mapping of residues that are predicted to be phosphorylated (green spheres), residues that are located at the interaction interface (blue spheres), and residues that are both phosphorylated and located at the interface (red) for glycoprotein E, NS3 (protease and helicase domain), and NS5. Structural overlay of HADDOCK-generated model of non-phosphorylated (blue) with (**B**) interface phosphorylated (red) and (**C**) completely phosphorylated (green) glycoprotein E, NS3 protease, NS3 helicase, and NS5. Note the near-complete overlay of the structures, suggesting the overall structural similarities upon phosphorylation. **(C)** The docking score (*left panel*), electrostatic energy (*middle panel*), and interaction energy (*right panel*) for the HADDOCK-derived structures of glycoprotein E: mAb ScFv EDE1 C10, NS3 protease: NS2B, NS3 helicase: NS5, and NS5:hSTAT2 that are non-phosphorylated (black), interface phosphorylated (dark grey), and completely phosphorylated (light grey). Note that the completely phosphorylated complexes show an unfavorable interaction energy compared to non-phosphorylated and interface phosphorylated complexes.

The structural and non-structural proteins of DENV-2 play crucial roles in mediating interactions with various host proteins to replicate efficiently in the cells and reach a mature state. Further, the neutralizing antibodies produced as a result of infection target these viral proteins to effectively neutralize their effect by transient interactions. For instance, the maturation of the virus occurs by the dissociation of prM protein from glycoprotein E when it translocates from the endoplasmic reticulum (ER) associated Golgi network through conformational changes [50]. Structurally composed of three domains (EDI, EDII, and EDIII), the glycoprotein E is known to interact with several cellular proteins (BiP, GRP-78, HSPA5, Rab5, CD-50, DC-SIGN2, and mannose receptor), and carbohydrate molecules, thereby mediating viral entry into the cell [51,52]. Further, the mapping of predicted phosphosites onto the known structures of structural and non-structural proteins showed that the phosphosites are localized throughout the proteins and hence, could play a critical role in protein-protein interactions (**Fig 4A**). For instance, glycoprotein E protein of DENV-2 is phosphorylated at multiple residues between residues 30–100, 110–125, 135–190, 200–280, and 295–480 that overlay and lie near the known protein-protein interaction sites (**Fig 4A**; **S1** **and** **S2 Figs**.). Among all these identified sites, extensive phosphorylation was identified in key interacting domains such as domain II (dimerization domain), domain III (receptor/antibody binding domain), and stem region (prM binding). Similarly, the other DENV-2 proteins, like NS1, NS3, and NS5, show predicted phosphosites in and around the host protein/antibody/viral protein interaction sites, with at least one phosphorylated residue involved in mediating these interactions (**Fig 4A**). For instance, the interaction of glycoprotein E with the scFv fragment of the antibody EDE1 C10 is mediated by the residue T155, which is predicted to be phosphorylated [53]. Also, the residue S304 predicted to be phosphorylated in NS1, residue Y23 in NS3 protease, T435 in NS3 helicase, and residues T314, S316, S318, and S319 in NS5, respectively, are involved in the interaction with interacting partners such as Ab 2B7, NS2B, NS5, and STAT2, respectively, highlighting the importance of phosphorylation in mediating their crucial interactions [54–57].

To identify the role of phosphorylation in the interaction of viral proteins with host/viral proteins and antibodies, we performed extensive molecular docking and molecular dynamics simulation studies. To this end, we have chosen the interactions of viral proteins that cover different interactions that include (i) antigen-antibody (glycoprotein E with mAb ScFv EDE1 C10), (ii) viral protein-viral protein (NS3 protease with NS2B and NS3 helicase with NS5), and (iii) host-pathogen (NS5 with hSTAT2) interactions. Further, to identify the differences in the phosphorylation at selective sites and all the sites of viral proteins, we have performed the docking studies in two ways: (i) docking the DENV-2 proteins with the residues that are phosphorylated only at the interaction interface, and (ii) docking the DENV-2 proteins where all the potential residues are phosphorylated, which may or may not occur all at the same time. Not surprisingly, the electrostatic potential is varied at the binding interface for these proteins upon phosphorylation due to the incorporation of negatively charged phosphate groups (**S13Fig**).

To benchmark whether our docking protocol is able to capture the crystal structure of the complexes, we performed the docking of native (non-phosphorylated) structures of the aforementioned complexes using HADDOCK. Our results show that the docked native structures overlay well with the crystal/cryo-EM structures with a backbone RMSD less than 0.72 Å for all the complexes confirming the performance of HADDOCK for biomolecular docking (**S14A** **and** **S14B Fig**). We then performed molecular docking of phosphorylated (at the binding interface and throughout the protein) viral proteins with their interacting partners using HADDOCK and the various parameters of the docking are summarized in **Table 2 and Table 3**. The structures of the phosphorylated viral proteins (glycoprotein E, NS3 protease, NS3 helicase, and NS5) in complex with their interacting partners overlay well with the non-phosphorylated complexes with a backbone RMSD in the range of 0.34–1.20 Å (**Fig 4B and 4C**). The comparison of docking scores of the non-phosphorylated and phosphorylated at the interface residues showed that the scores are nearly similar for all the complexes (**Fig 4D**, **Table 2 and 3**). For instance, the glycoprotein E in the presence and absence of phosphorylated interface residues showed similar docking scores of −74.21 ± 2.0 kcal/mol and −70.14 ± 3.9, respectively (**Table 2**). However, the docking scores were significantly reduced (with the glycoprotein E complex showing a positive docking score) when all the predicted PH sites were phosphorylated, suggesting that the restraint energy is unfavorable due to the introduction of a large number of negatively charged phosphorylated residues (**Fig 4D**, **S13 Fig**). This is also reflected in the interaction, wherein the introduction of negative charges leads to a largely unfavorable interaction energy, despite showing similar electrostatic energy (**Fig 4D**, **Table 2 and Table 3**).

**Table 2:** Structural statistics of HADDOCK-derived models generated for non-phosphorylated, interface phosphorylated, and completely phosphorylated DENV-2 proteins in complex with their interactors.

PH status	DENV proteins	HADDOCK-score (kcal/mol)	RMSD (Å)	N_struc_	E_desolv_ (kcal/mol)	E_elec_ (kcal/mol)	E_vdw_ (kcal/mol)	E_inter_ (kcal/mol)	BSA (Å²)
**NP**	**E**	−74.21 ± 2.0	1.31 ± 0.9	59	−9.12 ± 4.3	−186.71 ± 44.7	−28.78 ± 6.4	−205.21 ± 47.3	1024.75 ± 65.7
	**NS3 Protease**	−219.49 ± 44.1	1.21 ± 1.0	6	−8.34 ± 2.4	−505.44 ± 97.9	−117.55 ± 24.2	−548.08 ± 139.6	3172.50 ± 371.7
	**NS3 Helicase**	−254.15 ± 24.7	0.98 ± 0.6	18	15.03 ± 3.8	−868.90 ± 44.0	−133.55 ± 10.2	−620.84 ± 100.7	5210.77 ± 250.9
	**NS5**	−239.34 ± 11.5	1.83 ± 1.1	33	−14.86 ± 2.6	−806.07 ± 98.1	−93.05 ± 13	−601.32 ± 108.5	4349.38 ± 113.0
**IP**	**E**	−70.14 ± 3.9	0.84 ± 0.6	92	−0.11 ± 3.6	−302.49 ± 44.2	−19.57 ± 4.3	−221.67 ± 38.7	1019.68 ± 35.8
	**NS3 Protease**	−227.69 ± 45.3	1.05 ± 0.7	6	−2.04 ± 3.7	−619.02 ± 49.8	−118.85 ± 29.0	−567.83 ± 126.2	3262.75 ± 232.0
	**NS3 Helicase**	−221.8 ± 19.0	0.66 ± 0.4	25	18.79 ± 1.1	−832.66 ± 85.4	−134.25 ± 10.6	−365.06 ± 59.9	5147.40 ± 264.8
	**NS5**	−294.68 ± 7.6	0.69 ± 0.4	66	−23.56 ± 3.6	−754.16 ± 39.4	−125.75 ± 8.0	−825.32 ± 43.8	4954.09 ± 100.1
**CP**	**E**	43.16 ± 2.0	1.46 ± 0.9	19	9.06 ± 1.9	−332.44 ± 8.2	−15.32 ± 1.7	811.31 ± 16.5	984.72 ± 65.2
	**NS3 Protease**	−164.11 ± 32.8	0.69 ± 0.5	5	2.79 ± 3.3	−601.74 ± 71.1	−88.38 ± 13.7	−271.8 ± 143.5	3008.64 ± 203.1
	**NS3 Helicase**	−127.89 ± 36.5	0.92 ± 0.6	13	12.90 ± 5.1	−772.46 ± 110.9	−104.61 ± 16.3	306.09 ± 182.3	4450.13 ± 326.9
	**NS5**	−8.23 ± 29.3	1.3 ± 1.1	5	−10.09 ± 5.7	−716.79 ± 111.0	−54.68 ± 11.2	1227.58 ± 142.9	3460.31 ± 288.4

**Table 3:** dG of binding (kcal/mol) post HADDOCK docking.

Complex	NP	IP	CP
E-Ab	−37.65	−24.44	−31.85
NS3 protease-NS2B	−207.37	−247.99	−131.60
NS3 helicase-NS5	−19.45	−94.50	−133.86
NS5-STAT2	−143.68	−148.44	−44.63

To further characterize the differences between phosphorylated and non-phosphorylated complexes, the interacting regions were superimposed (**S15A-D Fig**.). In all cases, hydrogen bonds, π-π stacking, and salt bridges were seen stabilizing the complexes. For instance, the residues E147, E148, H149, D154, and D362 mediate the interaction of non-phosphorylated E protein with ScFv. However, when the interacting interface of E protein was phosphorylated, only H149, E147, and E148 were involved in the interaction with ScFv. Additionally, upon phosphorylation, Thr155, which was located in the binding site but not interacting with ScFv, was seen forming a hydrogen bond with Lys42 of ScFv (**S15A Fig**.), thereby stabilizing this interaction. Upon structural comparison of non-phosphorylated NS3 (protease and helicase domain) with phosphorylated complexes, an equal number of hydrogen bonds and salt bridges were seen in both cases, with similar residues seen involved in favorable interactions (**S15B** **and** **S15C Fig**.). The number of residues involved in the interaction of phosphorylated NS5 in the complex with hSTAT2 is less than in the non-phosphorylated complex (**S15D Fig**.). In all these cases, complete phosphorylation of viral proteins led to decreased interactions compared to non-phosphorylated viral proteins, resulting in weaker binding. Thus, when all the predicted phosphosites are phosphorylated, the interaction with their interacting partners becomes unfavorable, suggesting that all these predicted PH sites may not be phosphorylated simultaneously. Further, the phosphorylation event might favor or hinder the interaction of viral proteins with the antibodies/host proteins/viral proteins and, hence, will determine the outcome of their biological function. For example, weak interaction between phosphorylated E protein and prM could lead to the dissociation of prM, thereby leading to virus maturation. Further, the weak affinity between phosphorylated NS3 protease with NS2B or vice-versa might altogether abolish its activity, thereby preventing protease-mediated cleavage of viral polyprotein. The results of our docking studies also support the existing data where the hyperphosphorylation of NS5 has been shown to dissociate the NS3-NS5 complex [18]. Hence, our docking analyses show that the complete phosphorylation of viral proteins leads to reduced stability and could have significant biological implications.

### Effect of phosphorylation on the stability of glycoprotein E-mAb EDE1 C10 and NS5-hSTAT2 interactions of DENV-2 proteins

**Fig 5:**
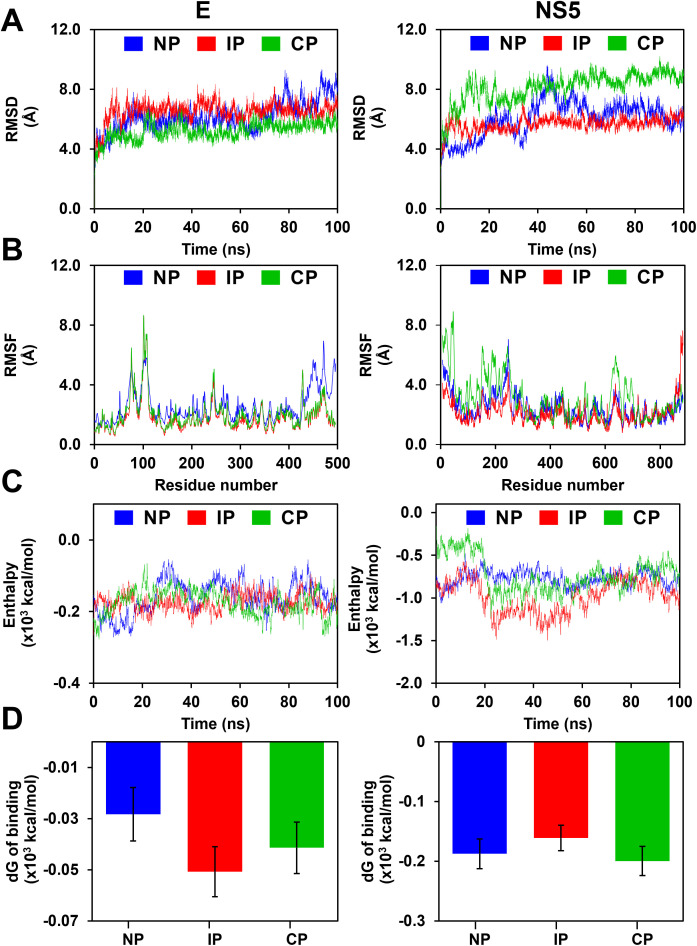
Molecular Dynamics simulations showing the role of phosphorylation in the stability of glycoprotein E: mAb EDE1 C10 and NS5:hSTAT2 interactions. **(A)** RMSD and **(B)** RMSF, (**C**) interaction energy, and (**D**) dG of binding plots for glycoprotein E: mAb EDE1 C10 (*left panel*) and NS5:hSTAT2 (*right panel*) complexes in the non-phosphorylated (blue), interface phosphorylated (red), and completely phosphorylated (green) states of the glycoprotein E and NS5 showing the stability of the complexes throughout the MD run. Note that the completely phosphorylated NS5 showed more significant fluctuations in all the plots, suggesting larger instability upon complete phosphorylation. The dG of the binding plot shows that the overall binding energy is very similar, wherein the negative impact of electrostatic energy due to large negative charges is compensated by the increased van der Waals energy.

To further test whether the phosphorylation of the predicted PH sites at the interface residues of the interaction and all the residues alters the stability of the complexes, we performed molecular dynamics (MD) simulations for the non-phosphorylated, phosphorylated at the interface and complete phosphorylated for two complexes, namely the glycoprotein E in complex with mAb ScFv EDE1 C10 and NS5 in complex with hSTAT2. The root mean square deviation (RMSD) was determined for the complexes at different phosphorylation states to delineate the changes in the backbone structure with respect to the initial structure. The mean backbone C^α^ RMSD of glycoprotein E-mAb EDE1 C10 complex is 6.51 ± 0.7 Å for interface phosphorylated (IP), 5.20 ± 0.6 Å for complete phosphorylated (CP) and 6.17 ± 1.0 Å for the non-phosphorylated complex suggesting that the complex is relatively stable throughout the MD run (**Fig 5A, *left panel*)**. On the other hand, the NS5-hSTAT2 complex showed larger fluctuations when it is completely phosphorylated (8.10 ± 1.0 Å) and is relatively stable in non-phosphorylated and interface phosphorylated complexes (IP- 5.67 ± 0.5 Å; NP-6.03 ± 1.2 Å) (**Fig 5A, *right panel***). In the case of glycoprotein E, complete phosphorylation leads to increased stability, whereas in NS5, complete phosphorylation leads to decreased stability compared to the interface phosphorylated and non-phosphorylated complexes. It is important to note that previous studies have shown that both the glycoprotein E and NS5 adopt multiple conformations in the non-phosphorylated state, which is also observed during the MD run [58,59]. This pattern is also observed in the root mean square fluctuation (RMSF) plots, which measure the average fluctuations per residue with respect to its mean position (**Fig 5B, *left and right panel*)**. Significantly, the complete phosphorylation of NS5 leads to more significant fluctuations compared to the non-phosphorylated and interface phosphorylated NS5, suggesting that the complete phosphorylation (and hence, the introduction of a large number of negatively charged residues) of NS5 leads to decreased stability of the overall complex.

MD simulations of the native, interface, and complete phosphorylation of glycoprotein E with antibody mAb ScFv ED1 C10 showed more favorable binding energetics for the interface phosphorylation than for the complete phosphorylation and non-phosphorylated complexes (**Fig 5C and 5D**). This trend is somewhat different from the HADDOCK docking score (Table 2), where unfavorable energetics are seen for complete phosphorylation of glycoprotein E-mAb ScFv EDE1 C10 complex. Structurally, most of the predicted phosphorylated residues were located at the surface of the glycoprotein E protein (except the residues T155, T176, T142, S145, T359, S363). Hence, when the glycoprotein E protein is completely phosphorylated, all the phosphorylated residues of the interface are not completely stabilized either by the presence of cationic residues in the vicinity or by solvent molecules like water and ions. On the contrary, upon interface phosphorylation of the residues (T155, S363) of the glycoprotein E protein and its complex with mAb ScFv EDE1 C10 is favored due to the presence of cationic residues (R54, H149) in the vicinity and favorable interactions due to water and ions (**S16A**, **S16B****, and** **S16C Figs****.).** In the case of NS5-STAT2 complex MD simulations, the calculated free energy of the complex (**Fig 5D**) for the interface phosphorylation complex is seen to be less favored (**Fig 5C and 5D**) compared to the native and complete phosphorylated NS5 complex. Taken together, overall RMSD, per residue fluctuations (RMSF), and interaction energies of the phosphorylated complex showed more fluctuations than the native non-phosphorylated complex (**Fig 5),** suggesting the possibility of dynamic instability of the phosphorylated complex. This could be due to the presence of many phosphorylated residues at the surface of NS5 and their continuous interactions with surface water molecules. However, considering that the possibility of heavy phosphorylation and stabilization by STAT2 and solvent molecules is improbable in the cellular conditions, phosphorylation of NS5-STAT2 may be less probable and viable for viral survival.

### Conservation of phosphosites across the different circulating strains of Dengue serotypes

Lastly, to test whether the phosphosites are conserved across circulating strains of different dengue serotypes, we performed extensive sequence analysis of these strains and compared them with the predicted phosphosites of DENV-2. To this end, extensive sequence mining was conducted using the NCBI Virus database, and a comprehensive alignment of all curated DENV protein sequences across serotypes was generated. Most importantly, the sequences of glycoprotein E (~9900), NS5 (~450), NS1 (~294), and NS3 (~136) are well-curated in the NCBI database and are therefore compared for phosphosite conservation across DENV-2 serotypes. Our results show that the number of phosphosites that are conserved across the four serotypes (**Fig 2**, **S1**, **S2**, **S3**, **S4**, **S5**, **S6**, **S7**, **S8****, and** **S9A Figs**.) are highly conserved across the circulating strains that includes 11 in glycoprotein E, nine in NS1, four in NS3, and 22 in NS5 (**S17**, **S18**, **S19****, and** **S20 Figs**) despite having differences in the loop sizes in glycoprotein E, NS1, NS3, and NS5 across different serotypes. Thus, our results suggest that the phosphosites are highly conserved across circulating strains of DENV serotypes, reiterating our findings on flaviviruses; hence, targeting the phosphorylation mechanism is a viable approach for developing therapeutics to combat DENV infection.

## Discussion

Phosphorylation of proteins by the kinases plays a critical role in key cellular processes like cell division, signal transduction, protein synthesis, and metabolism [60]. Upon infecting the host cells, viruses have evolved numerous mechanisms for efficient replication and an exponential increase in their copy number, one of the most efficient being the hijacking of phosphorylation signaling for their benefit [61,62]. Dengue virus is one of the major flaviviruses, replicating in the cell by hijacking the host cell’s machinery to produce structural and non-structural viral proteins. However, it is unknown how the phosphorylation signaling is affected and how the Dengue virus replicates using phosphorylation. Hence, it is essential to understand the phosphorylation status of the Dengue virus proteins. Here, we used bioinformatic tools and structural approaches to identify the phosphosites of Dengue virus proteins and their role in protein-protein interactions. Our results show that both the structural and non-structural proteins of the Dengue virus have higher propensities (5–9% of total residues) for phosphorylation upon infection.

Several studies have previously explored the role of phosphorylation in viral infection. For instance, it has been shown that MAPKs like JNK, ERK1/2, and p38 are activated in macrophages upon DENV infection, and the inhibitors of the MAPK pathway reduce the viral activity [63–65]. In another study, inhibition of pyruvate kinase M2 (PKM2), which is involved in glycolysis and is required for DENV replication, showed a small but significant decrease in DENV infection levels and viral output [66]. Previous studies have also demonstrated that the knockdown of receptor tyrosine kinases (RTKs) like EPHB4, ERBB2, FGFR2, or IGF1R reduces DENV infection in hepatocytes [67]. Apart from phosphorylation of self-proteins in case of dengue infection, the dengue virus also induces phosphorylation of host proteins. For example, phosphorylated Vimentin was found to interact with NS4A, which leads to cytoskeletal reorganization, thereby causing anchorage of the replication complex in ER [68]. Although the mechanism by which NS4A induces phosphorylation of Vimentin is not known, one might hypothesize hijacking of the cellular signaling pathway leading to phosphorylation of Vimentin, as seen in the case of WNV E DIII protein interaction with αvβ3 integrin, where E DIII- αvβ3 integrin interaction induces phosphorylation of focal adhesion kinase, which triggers outside-in signaling pathway, which in turn helps virus internalization [69]. Additionally, the WNV C protein undergoes phosphorylation by PKC, which is imperative for interacting with importin α and HDM2 protein [70]. In the Yellow Fever Virus (YFV), phosphorylation by CK1 at 56S (conserved across flaviviruses) of NS5 methyltransferase inhibits its methyltransferase activity for the 2’-O- methylation reaction that requires the hydroxyl group of serine, which upon phosphorylation, is replaced with a phosphate group [71].

We show that nearly 14 kinases play crucial roles in the phosphorylation of DENV proteins and alter the interaction profiles with various viral/antibody/host proteins. Among the kinases, we show that the AGC kinase family (Protein Kinase A, G, and C) plays a significant role in the phosphorylation of most Dengue viral proteins. We also show that most of the predicted phosphosites are localized in the solvent-exposed loop regions, as observed for most phosphorylated proteins. Most importantly, we established that the predicted phosphosites are highly conserved across the multiple DENV serotypes as well as other flaviviruses (with >100 sequences of flaviviruses analyzed) like West Nile virus (WNV), tick-borne encephalitis virus (TEV), yellow fever virus, and Zika virus, among others. Thus, we showed that these conserved phosphosites are evolutionarily conserved across the various flaviviruses. Hence, the development of antivirals that function as kinase activators could aid in the development of novel therapeutics to neutralize not only dengue viral infection but also other flavivirus infections.

As a result of viral infection, the host immune system produces antibodies to neutralize the foreign pathogens. Hence, it is imperative that the phosphorylation status of the viral proteins could play crucial roles in the interaction of viral proteins with antibodies. Most importantly, among all the viral proteins of Dengue, glycoprotein E is the target of many neutralizing antibodies and plays a crucial role in viral entry. Here, we tested the effect of phosphorylation of glycoprotein E with antibodies. The interaction of glycoprotein E with antibodies is weakened in the docking studies; however, it shows increased stability during MD simulations. This is plausible because glycoprotein E adopts multiple conformations in solution, and phosphorylation forms a stable complex, efficiently binding with the antibodies, leading to the efficient neutralization of viral infection.

The viruses replicate and mature by interacting with the various structural/non-structural proteins. For instance, NS3 helicase and protease domains interact with the NS5 and NS2B, respectively, to form the RNA replicase complex and determine the outcome of viral replication. Our study shows that the phosphorylation of the NS3 helicase or protease domain destabilizes its interaction with NS5 and NS2B, potentially inhibiting viral replication. Hence, it is evident that the phosphorylation of viral proteins could alter these interactions, potentially hampering the virus lifecycle and aiding in the neutralization of viral infection.

In flaviviruses, non-structural proteins are known to recruit host proteins such as Lunapark (LNP) protein, Reticulon 3.1A, Transmembrane Protein 41B (TMEM41B), and Vacuole membrane protein 1 (VMP1), Receptor for Activated C Kinase 1 (RACK1) protein, and ER membrane complex (EMC) to achieve stability, functionality, ER remodeling and anchorage of replication complex to ER [72–78]. Further, the structural and non-structural proteins are involved in multiple transient interactions with host proteins throughout the virus lifecycle [79,80]. For instance, NS1 (that exists as a monomer in endoplasmic reticulum, homodimer with membrane and a hexamer when secreted outside the infected cells) is known to perform different functions like its dimer is known to bind with NS4A, NS4B, 2K, and viral RNA, which leads to formation of viral replicase complex in infected cell cytoplasm, in addition to, eliciting a strong immune response thereby shielding other immunogenic dengue proteins. Here, we show that the phosphorylation of dengue viral proteins like NS5 also affects the interaction with host proteins like hSTAT2. Our results show that the phosphorylation of the NS5 (both interface phosphorylation and complete phosphorylation) destabilizes the NS5-hSTAT2 complex. NS5 forms a complex with STAT2 along with ubiquitin ligase E3 recognin 4 (UBR4), which is responsible for ubiquitination and degradation of STAT2 [81,82]. The complex of STAT2, NS5, and UBR4 sends the signal for ubiquitination, which degrades the STAT2, which in turn inhibits the type 1 IFN signaling pathway of the host immune response. However, phosphorylation of NS5 leads to the destabilization of the NS5-hSTAT2 interaction and, hence, will lead to the proper functioning of STAT2, leading to the efficient neutralization of viral infection. Thus, developing small molecule activators mimicking the phosphorylation of NS5 could aid in treating dengue viral infection.

In summary, phosphorylation is critical in interacting with host/viral proteins and antibodies. We also identify the key phosphosites and the kinases that phosphorylate them across the DENV serotypes. We have also shown that the phosphosites conserved across the DENV serotypes are indeed conserved across the other flaviviruses. Our study could help in future work that is focused on elucidating the role of phosphosites in viral pathogenesis using *in vitro* and *in vivo* approaches.

## Supporting information

S1 Fig Overview of PH sites in Dengue virus serotype 2 viral proteins: Capsid (C), prM, glycoprotein E, NS1, NS2A. *Left panel* – Lollipop graphs showing the phosphosites predicted using NetPhos3.1; *Middle panel* – The best hit kinases for each of the predicted phosphosites and, *Right panel* – the distribution of secondary structural elements in the viral proteins (pink-loop; cyan-beta turns; orange-extended strand; blue- alpha helix) analyzed using SOPMA secondary structure prediction software. Also indicated are the total number of phosphosites that are present in a particular secondary structure.
(TIF)

S2 Fig Overview of PH sites in Dengue virus serotype 2 viral proteins: NS2B, NS3, NS4A, NS4B, and NS5. *Left panel* – Lollipop graphs showing the phosphosites predicted using NetPhos3.1; *Middle panel* – The best hit kinases for each of the predicted phosphosites and, *Right panel* – the distribution of secondary structural elements in the viral proteins (pink-loop; cyan-beta turns; orange-extended strand; blue- alpha helix) analyzed using SOPMA secondary structure prediction software. Also indicated are the total number of phosphosites that are present in a particular secondary structure.
(TIF)

S3 Fig Overview of phosphosites in Dengue virus serotype 1 viral proteins: Capsid (C), prM, glycoprotein E, NS1, NS2A. *Left panel* – Lollipop graphs showing the phosphosites predicted using NetPhos3.1; *Middle panel* – The best hit kinases for each of the predicted phosphosites and, *Right panel* – the distribution of secondary structural elements in the viral proteins (pink-loop; cyan-beta turns; orange-extended strand; blue- alpha helix) analyzed using SOPMA secondary structure prediction software. Also indicated are the total number of phosphosites that are present in a particular secondary structure.
(TIF)

S4 Fig Overview of phosphosites in Dengue virus serotype 1 viral proteins: NS2B, NS3, NS4A, NS4B, and NS5. *Left panel* – Lollipop graphs showing the phosphosites predicted using NetPhos3.1; *Middle panel* – The best hit kinases for each of the predicted phosphosites and, *Right panel* – the distribution of secondary structural elements in the viral proteins (pink-loop; cyan-beta turns; orange-extended strand; blue- alpha helix) analyzed using SOPMA secondary structure prediction software. Also indicated are the total number of phosphosites that are present in a particular secondary structure.
(TIF)

S5 Fig Overview of phosphosites in Dengue virus serotype 3 viral proteins: Capsid (C), prM, glycoprotein E, NS1, NS2A. *Left panel* – Lollipop graphs showing the phosphosites predicted using NetPhos3.1; *Middle panel* – The best hit kinases for each of the predicted phosphosites and, *Right panel* – the distribution of secondary structural elements in the viral proteins (pink-loop; cyan-beta turns; orange-extended strand; blue- alpha helix) analyzed using SOPMA secondary structure prediction software. Also indicated are the total number of phosphosites that are present in a particular secondary structure.
(TIF)

S6 Fig Overview of phosphosites in Dengue virus serotype 3 viral proteins: NS2B, NS3, NS4A, NS4B, and NS5. *Left panel* – Lollipop graphs showing the phosphosites predicted using NetPhos3.1; *Middle panel* – The best hit kinases for each of the predicted phosphosites and, *Right panel* – the distribution of secondary structural elements in the viral proteins (pink-loop; cyan-beta turns; orange-extended strand; blue- alpha helix) analyzed using SOPMA secondary structure prediction software. Also indicated are the total number of phosphosites that are present in a particular secondary structure.
(TIF)

S7 Fig Overview of phosphosites in Dengue virus serotype 4 viral proteins: Capsid (C), prM, glycoprotein E, NS1, NS2A. *Left panel* – Lollipop graphs showing the phosphosites predicted using NetPhos3.1; *Middle panel* – The best hit kinases for each of the predicted phosphosites and, *Right panel* – the distribution of secondary structural elements in the viral proteins (pink-loop; cyan-beta turns; orange-extended strand; blue- alpha helix) analyzed using SOPMA secondary structure prediction software. Also indicated are the total number of phosphosites that are present in a particular secondary structure.
(TIF)

S8 Fig Overview of phosphosites in Dengue virus serotype 4 viral proteins: NS2B, NS3, NS4A, NS4B, and NS5. *Left panel* – Lollipop graphs showing the phosphosites predicted using NetPhos3.1; *Middle panel* – The best hit kinases for each of the predicted phosphosites and, *Right panel* – the distribution of secondary structural elements in the viral proteins (pink-loop; cyan-beta turns; orange-extended strand; blue- alpha helix) analyzed using SOPMA secondary structure prediction software. Also indicated are the total number of phosphosites that are present in a particular secondary structure.
(TIF)

S9 Fig Comparison of PH sites of DENV-2 with other DENV serotypes. (A) Venn diagram showing the predicted phosphosites for the C, prM, NS2A, NS2B, NS4A, and NS4B in Dengue virus serotypes 1, 2, 3, and 4. Note the number of overlapping phosphosites across multiple DENV serotypes. (B) Web logo showing the phosphorylation cluster across the sequences in the C, prM, NS2A, and NS4B proteins of flaviviruses. The sequence motifs near the conserved phosphosites in DENV serotypes are shown. The conserved phosphosites from the DENV serotypes are highlighted in an orange box, and the bits show the degree of conservation across flaviviruses (a bit value of 4 represents a highly conserved residue, whereas a value of 1 shows the least conservation at that position).
(TIF)

S10 Fig Multiple Sequence Alignment of Dengue virus proteins. Multiple sequence alignment of Dengue viral proteins (serotypes 1, 2, 3, and 4) Capsid, prM, glycoprotein E, NS1.
(TIF)

S11 Fig Multiple Sequence Alignment of Dengue virus proteins. Multiple sequence alignment of Dengue viral proteins (serotypes 1, 2, 3, and 4) NS2A, NS2B, NS3, and NS4A.
(TIF)

S12 Fig Multiple Sequence Alignment of Dengue virus proteins. Multiple sequence alignment of Dengue viral proteins (serotypes 1, 2, 3, and 4) NS4B, and NS5.
(TIF)

S13 Fig Surface electrostatic potential of Dengue viral proteins. Surface electrostatic potential of Dengue viral proteins (A) glycoprotein E (PDB ID: 7CTH), (B) NS3 protease (PDB ID: 2FOM), (C) NS3 helicase (PDB ID: 8GZQ), and (D) NS5 (PDB ID: 8T12) in non-phosphorylated (left panel) and interface phosphorylated (right panel) states. The interaction interface is highlighted in a cyan circle, and the changes in electrostatic potential are highlighted in the green box. Blue represents positive charge potential, and red represents negative charge potential. The inputs for computing the electrostatic potential using the Advanced Poisson-Boltzmann Solver (APBS) were generated with the PDB2PQR tool.
(TIF)

S14 Fig Structural comparison of PDB structures with HADDOCK-generated models. (A) Overlay of non-phosphorylated crystal structure (cyan) and HADDOCK docked (blue) of glycoprotein E: mAb ScFv EDE1 C10 (PDB ID: 7CTH), NS3 protease: NS2B (PDB ID: 2FOM), NS3 helicase: NS5 (PDB ID: 8GZQ) and NS5:hSTAT2 (PDB ID: 8T12). Note that all the structures overlay well suggesting that HADDOCK generated models are similar to that of the crystal structures. (B) The C^α^ backbone RMSD of the overlay of PDB structures with interface/completely phosphorylated DENV proteins shows that the structures generated for phosphorylated states are very similar to the non-phosphorylated DENV proteins.
(TIF)

S15 Fig Interface mapping of Dengue proteins in complex with their interactors. Surface and stick representation of interface in (A) glycoprotein E in complex with the scFv fragment of the mAb EDE1 C10, (B) NS3 protease in complex with NS2B, (C) NS3 helicase in complex with NS5, (D) NS5 in complex with human STAT2. The non-phosphorylated residues of dengue proteins are shown in the thin pink ball and stick model, whereas the phosphorylated residues are shown in the thick light pink ball and stick model. The residues of the interacting proteins of non-phosphorylated protein are shown in a thin royal blue ball and stick model, whereas the residues associated with phosphorylated proteins are shown in thick sky-blue ball and stick model. Also shown is the surface of Dengue proteins (light golden – phosphorylated and dark golden – non-phosphorylated) and interacting proteins (light cyan – phosphorylated and dark cyan – non-phosphorylated). The interacting residues are also labeled. Note that the NS3 protease lacks phosphorylation sites at the interface but shows differences in the interaction due to the phosphorylation of other residues due to long-range allosteric effects.
(TIF)

S16 Fig Solvent accessible surface area analysis for the DENV-2 proteins with antibodies and host proteins. (A) Solvent accessible surface area (SASA) plot for the glycoprotein E and NS5 in the glycoprotein E: mAb ScFv EDE1 C10 (*left panel*) and NS5:hSTAT2 (*right panel*) for the last 50 ns of the MD run. The complexes in the non-phosphorylated (blue), interface phosphorylated (red), and completely phosphorylated (green) states of the glycoprotein E and NS5 showed less variability of the SASA during the course of the MD run. Plot showing the residues that are involved in various types of interactions like the π-cation interaction (blue), hydrogen bond (purple), salt bridges (orange), water bridges (cyan), and hydrophobic (green) interactions for the (B) glycoprotein E: mAb ScFv EDE1 C10 (*left panel*) and (C) NS5:hSTAT2. Note that both these complexes have higher interaction fractions for the interaction of residues with the water molecules.
(TIF)

S17 Fig Sequence comparison and phosphosite conservation across the circulating strains of DENV serotypes. Sequence alignment of consensus sequence of circulating strains of DENV serotypes with DENV2 sequence for Envelope glycoprotein E. The conserved phosphosites in DENV-2 are highlighted in magenta box. Note that the predicted phosphosites are highly conserved across the circulating strains of DENV serotypes.
(TIF)

S18 Fig Sequence comparison and phosphosite conservation across the circulating strains of DENV serotypes. Sequence alignment of consensus sequence of circulating strains of DENV serotypes with DENV2 sequence for NS1.The conserved phosphosites in DENV-2 are highlighted in magenta box. Note that the predicted phosphosites are highly conserved across the circulating strains of DENV serotypes.
(TIF)

S19 Fig Sequence comparison and phosphosite conservation across the circulating strains of DENV serotypes. Sequence alignment of consensus sequence of circulating strains of DENV serotypes with DENV2 sequence for NS3. The conserved phosphosites in DENV-2 are highlighted in magenta box. Note that the predicted phosphosites are highly conserved across the circulating strains of DENV serotypes.
(TIF)

S20 Fig Sequence comparison and phosphosite conservation across the circulating strains of DENV serotypes. Sequence alignment of consensus sequence of circulating strains of DENV serotypes with DENV2 sequence for NS5. The conserved phosphosites in DENV-2 are highlighted in magenta box. Note that the predicted phosphosites are highly conserved across the circulating strains of DENV serotypes.
(TIF)

S1 Table Distribution of phosphosites across the various viral proteins of DENV1. (DOCX)

S2 Table Distribution of phosphosites across the various viral proteins of DENV3. (DOCX)

S3 Table Distribution of phosphosites across the various viral proteins of DENV4. (DOCX)

S4 Table Accession IDs for the C protein sequences used in the phosphosite evolutionary conservation analysis. (DOCX)

S5 Table Accession IDs for the prM protein sequences used in the phosphosite evolutionary conservation analysis. (DOCX)

S6 Table Accession IDs for the E protein sequences used in the phosphosite evolutionary conservation analysis. (DOCX)

S7 Table Accession IDs for the NS1 protein sequences used in the phosphosite evolutionary conservation analysis. (DOCX)

S8 Table Accession IDs for the NS2A protein sequences used in the phosphosite evolutionary conservation analysis. (DOCX)

S9 Table Accession IDs for the NS3 protein sequences used in the phosphosite evolutionary conservation analysis. (DOCX)

S10 Table Accession IDs of the NS4B protein sequences used in the phosphosite evolutionary conservation analysis. (DOCX)

S11 Table Accession IDs for the NS5 protein sequences used in the phosphosite evolutionary conservation analysis. (DOCX)
