# Supplementary figures and images for: Phosphorylation landscape of dengue virus proteins and their implications in protein-protein interactions
Source: PLoS One. 2026 May 12;21(5):e0345872. doi: 10.1371/journal.pone.0345872 (PMC13166905; doi:10.1371/journal.pone.0345872)

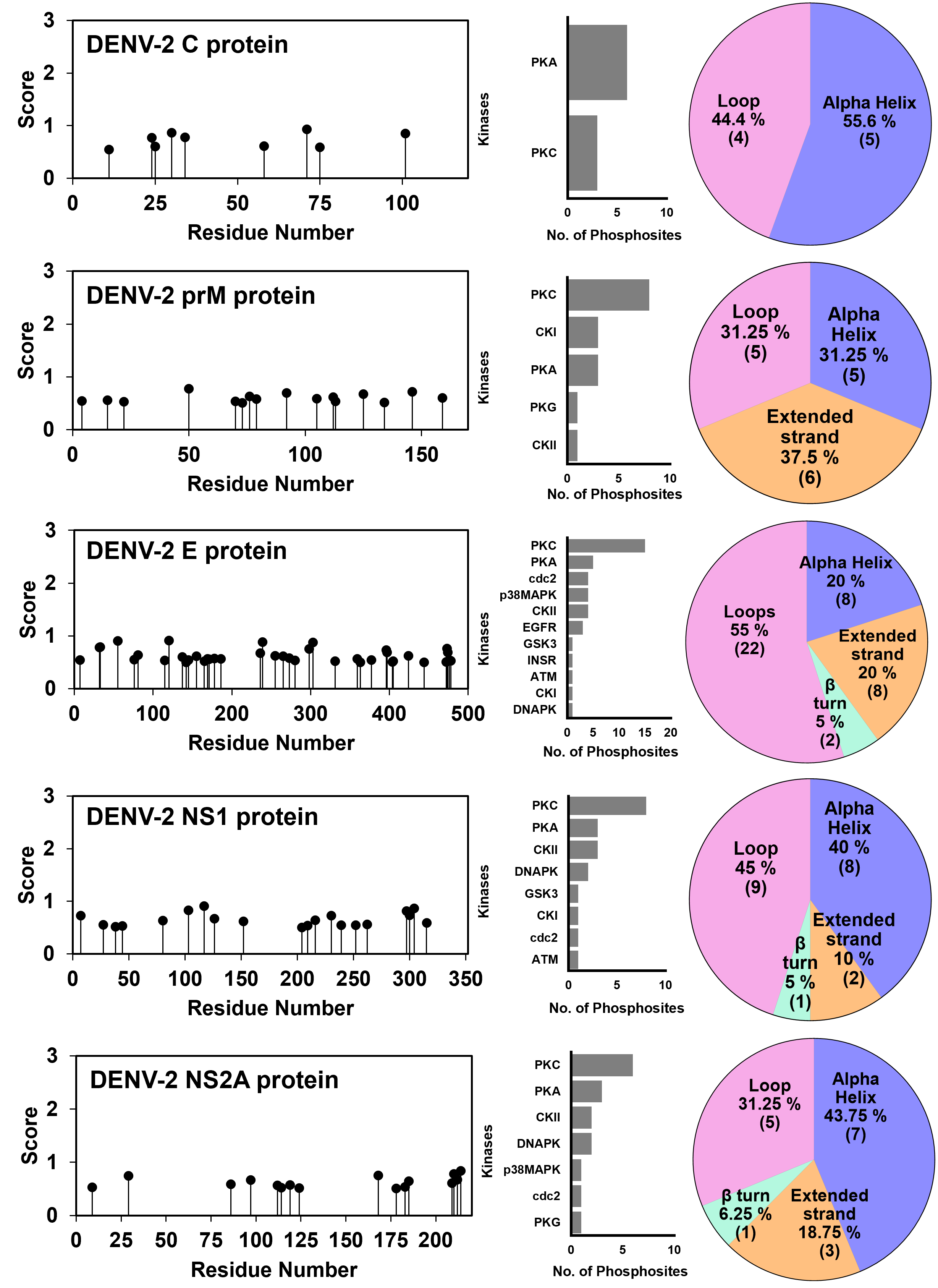

Supplement: S1 Fig — Left panel – Lollipop graphs showing the phosphosites predicted using NetPhos3.1; Middle panel – The best hit kinases for each of the predicted phosphosites and, Right panel – the distribution of secondary structural elements in the viral proteins (pink-loop; cyan-beta turns; orange-extended strand; blue- alpha helix) analyzed using SOPMA secondary structure prediction software. Also indicated are the total number of phosphosites that are present in a particular secondary structure. (TIF) [file pone.0345872.s001.tif]

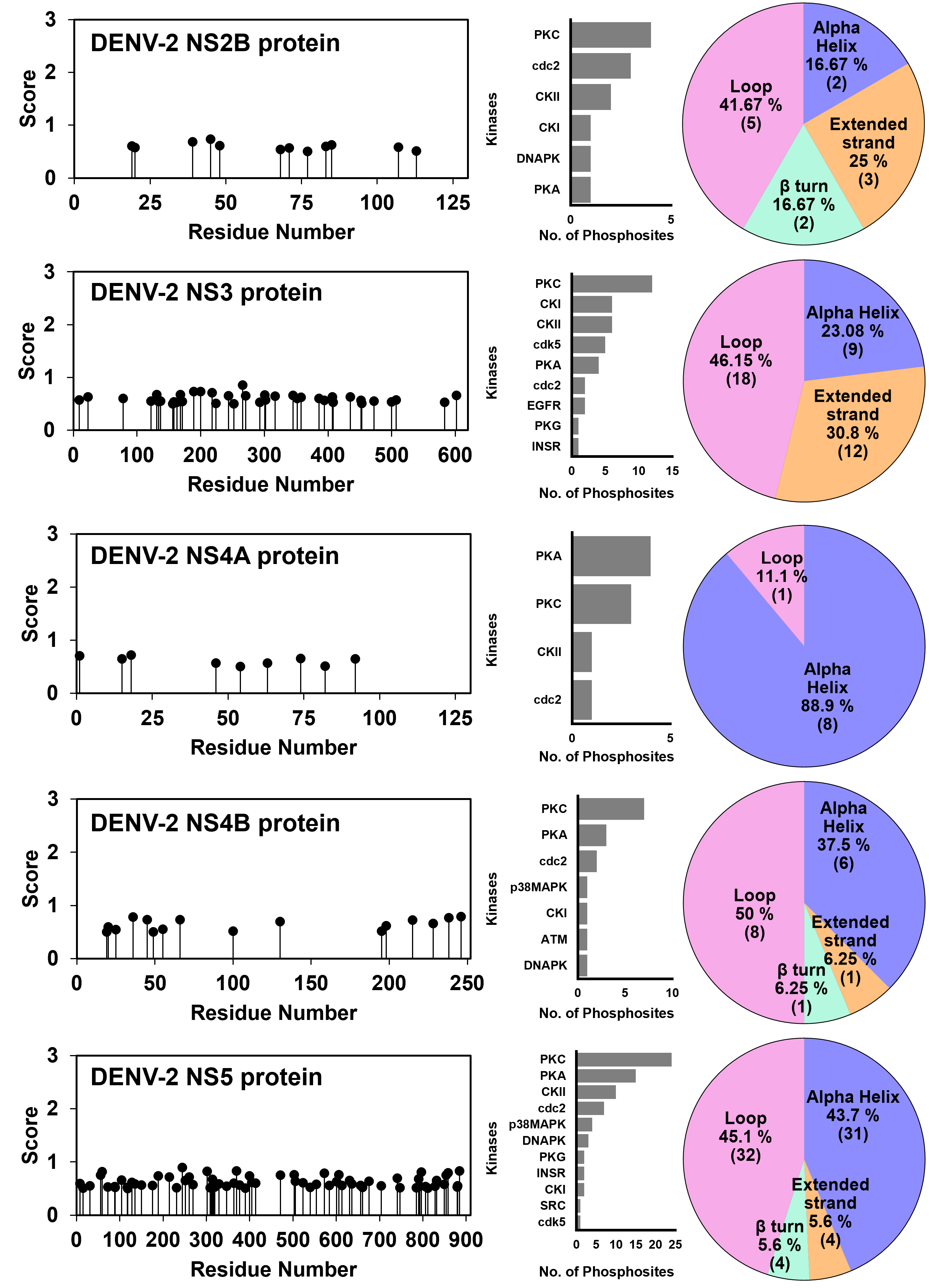

Supplement: S2 Fig — Left panel – Lollipop graphs showing the phosphosites predicted using NetPhos3.1; Middle panel – The best hit kinases for each of the predicted phosphosites and, Right panel – the distribution of secondary structural elements in the viral proteins (pink-loop; cyan-beta turns; orange-extended strand; blue- alpha helix) analyzed using SOPMA secondary structure prediction software. Also indicated are the total number of phosphosites that are present in a particular secondary structure. (TIF) [file pone.0345872.s002.tif]

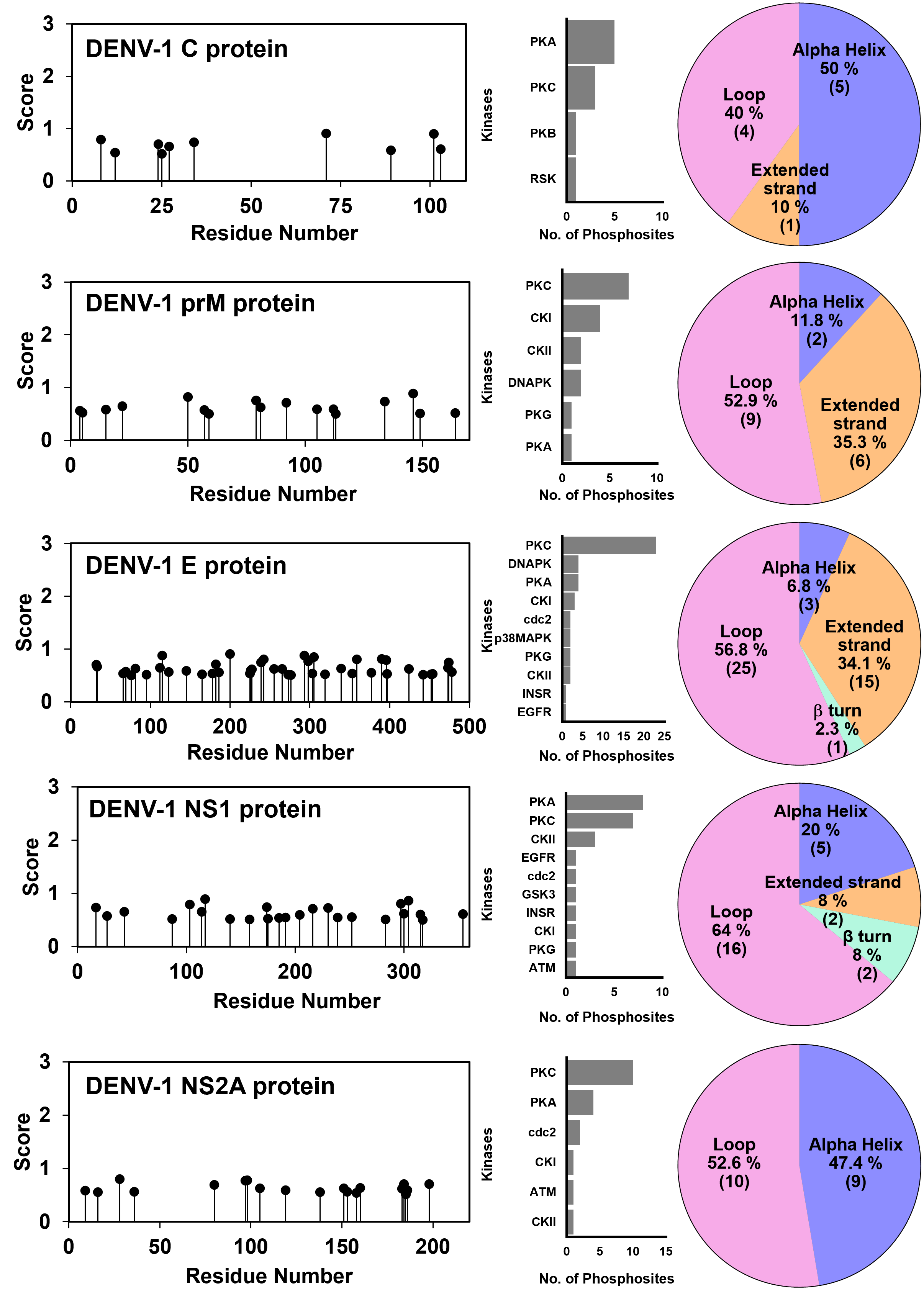

Supplement: S3 Fig — Left panel – Lollipop graphs showing the phosphosites predicted using NetPhos3.1; Middle panel – The best hit kinases for each of the predicted phosphosites and, Right panel – the distribution of secondary structural elements in the viral proteins (pink-loop; cyan-beta turns; orange-extended strand; blue- alpha helix) analyzed using SOPMA secondary structure prediction software. Also indicated are the total number of phosphosites that are present in a particular secondary structure. (TIF) [file pone.0345872.s003.tif]

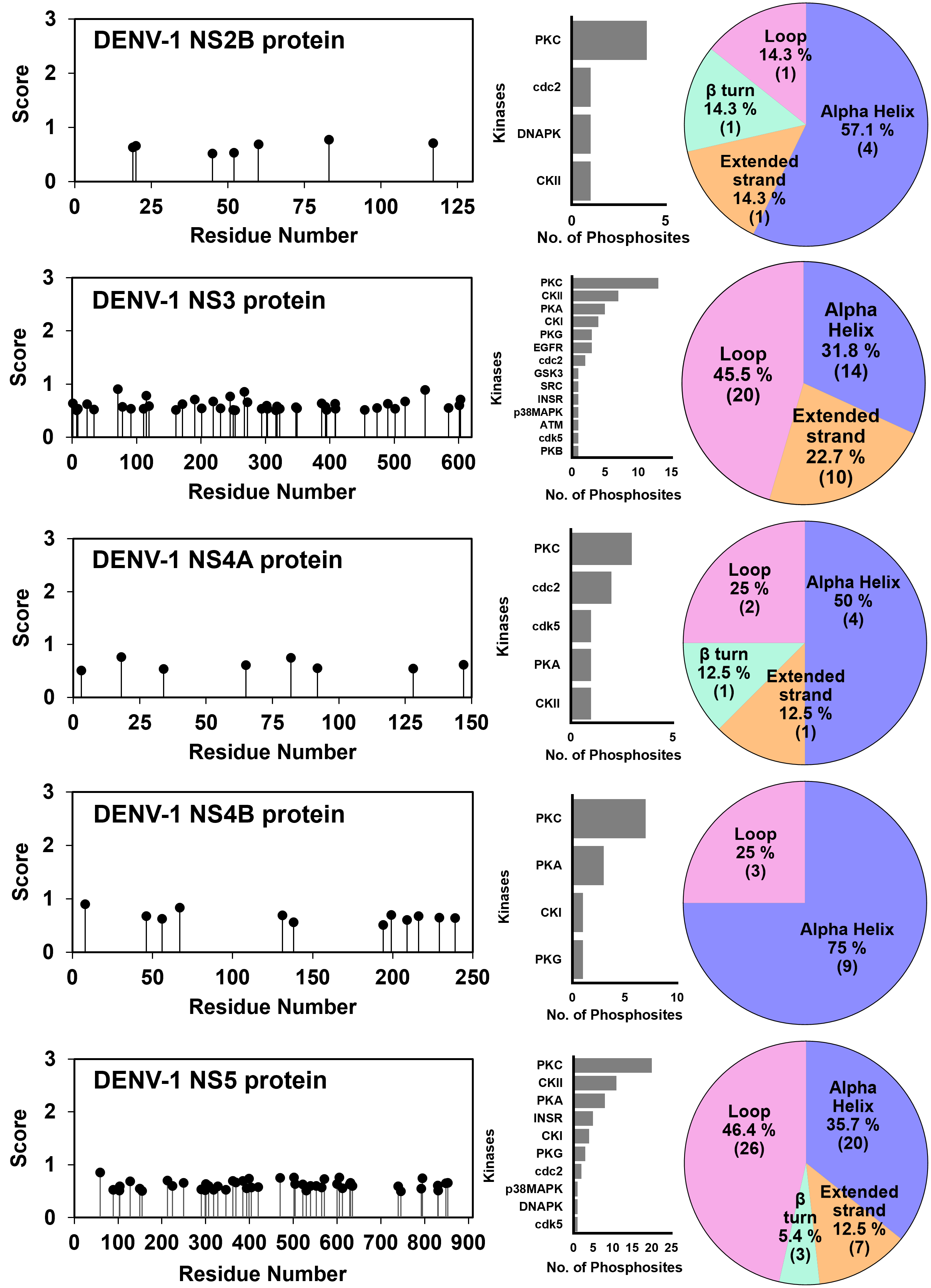

Supplement: S4 Fig — Left panel – Lollipop graphs showing the phosphosites predicted using NetPhos3.1; Middle panel – The best hit kinases for each of the predicted phosphosites and, Right panel – the distribution of secondary structural elements in the viral proteins (pink-loop; cyan-beta turns; orange-extended strand; blue- alpha helix) analyzed using SOPMA secondary structure prediction software. Also indicated are the total number of phosphosites that are present in a particular secondary structure. (TIF) [file pone.0345872.s004.tif]

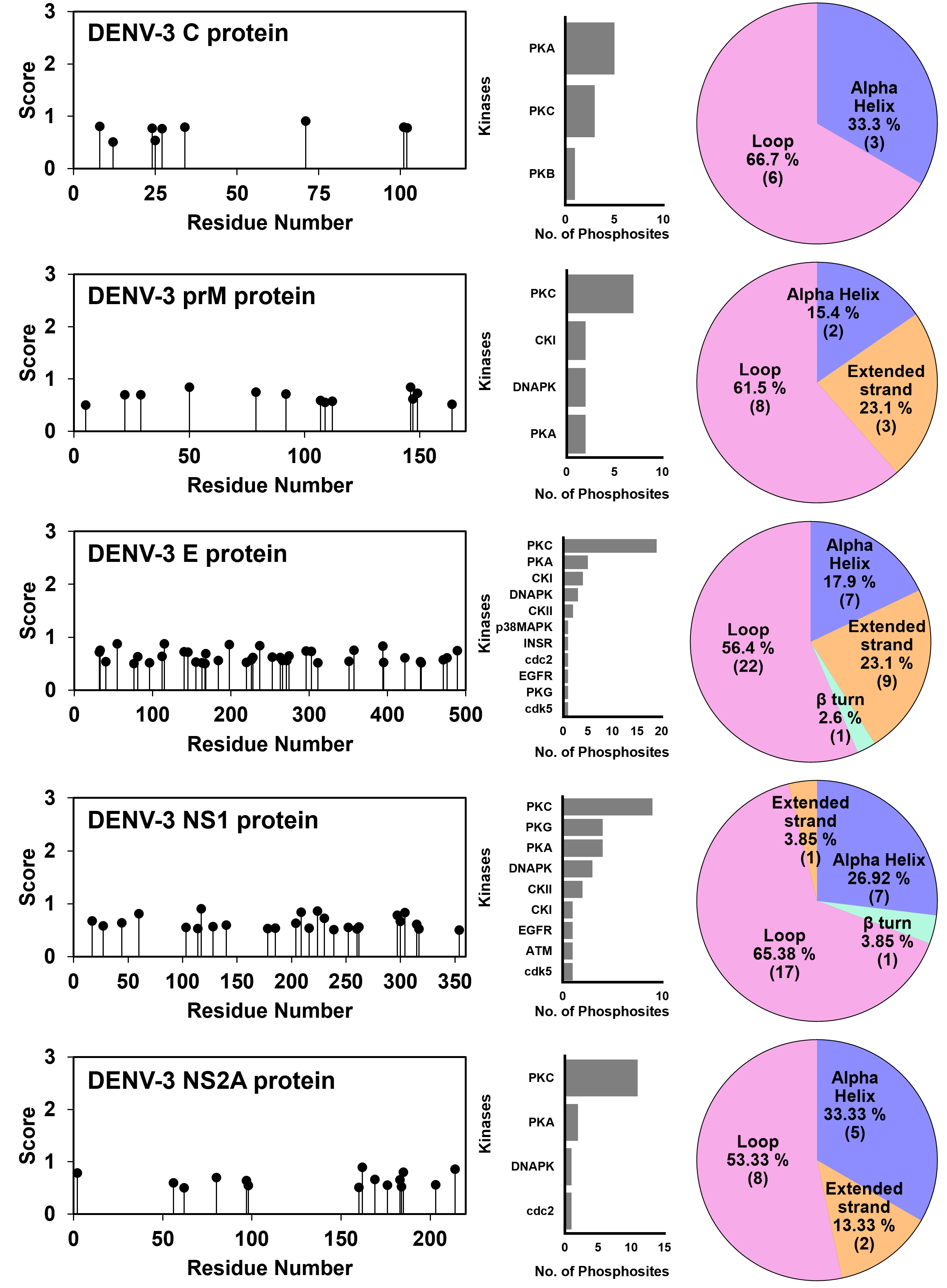

Supplement: S5 Fig — Left panel – Lollipop graphs showing the phosphosites predicted using NetPhos3.1; Middle panel – The best hit kinases for each of the predicted phosphosites and, Right panel – the distribution of secondary structural elements in the viral proteins (pink-loop; cyan-beta turns; orange-extended strand; blue- alpha helix) analyzed using SOPMA secondary structure prediction software. Also indicated are the total number of phosphosites that are present in a particular secondary structure. (TIF) [file pone.0345872.s005.tif]

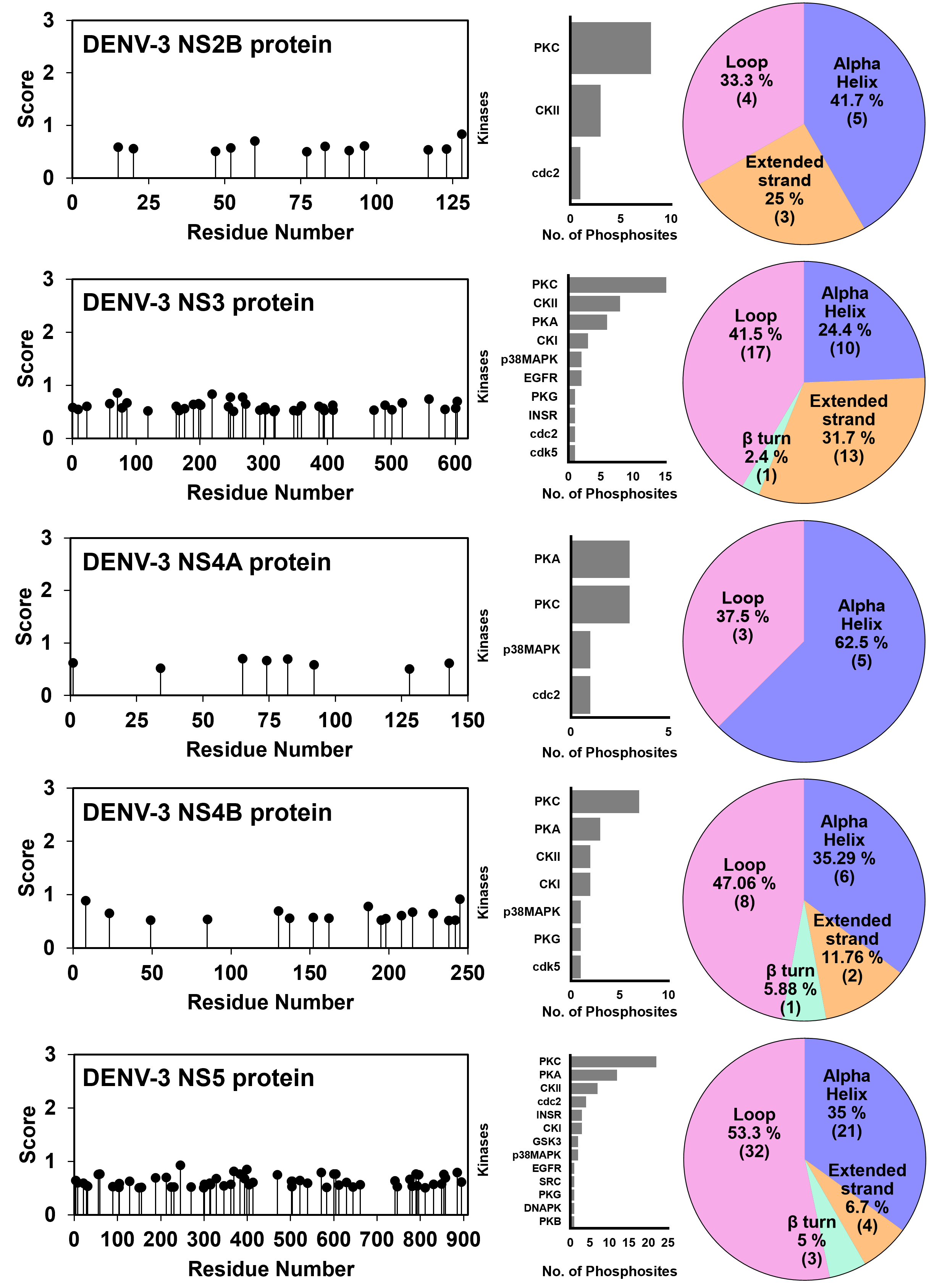

Supplement: S6 Fig — Left panel – Lollipop graphs showing the phosphosites predicted using NetPhos3.1; Middle panel – The best hit kinases for each of the predicted phosphosites and, Right panel – the distribution of secondary structural elements in the viral proteins (pink-loop; cyan-beta turns; orange-extended strand; blue- alpha helix) analyzed using SOPMA secondary structure prediction software. Also indicated are the total number of phosphosites that are present in a particular secondary structure. (TIF) [file pone.0345872.s006.tif]

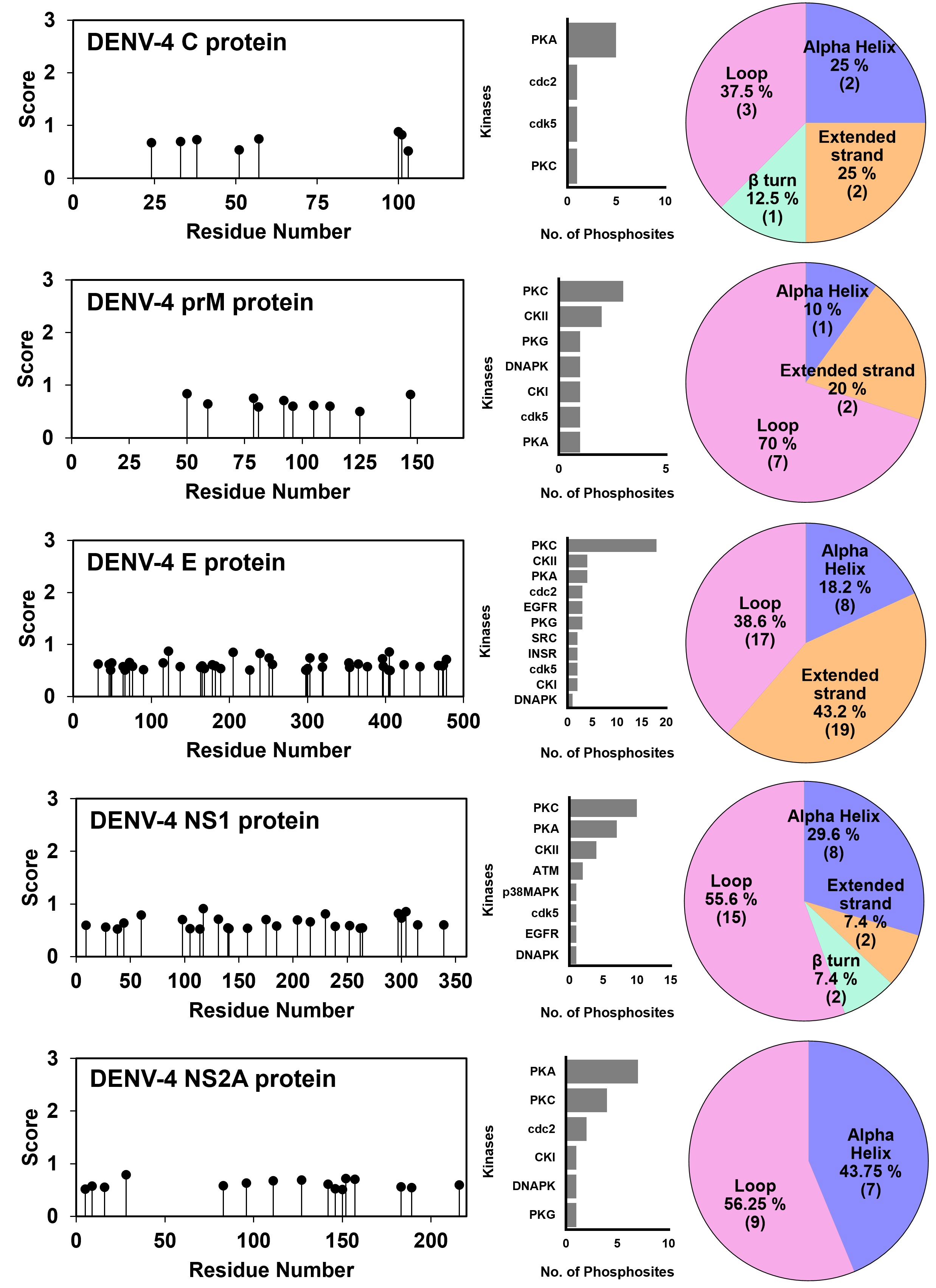

Supplement: S7 Fig — Left panel – Lollipop graphs showing the phosphosites predicted using NetPhos3.1; Middle panel – The best hit kinases for each of the predicted phosphosites and, Right panel – the distribution of secondary structural elements in the viral proteins (pink-loop; cyan-beta turns; orange-extended strand; blue- alpha helix) analyzed using SOPMA secondary structure prediction software. Also indicated are the total number of phosphosites that are present in a particular secondary structure. (TIF) [file pone.0345872.s007.tif]

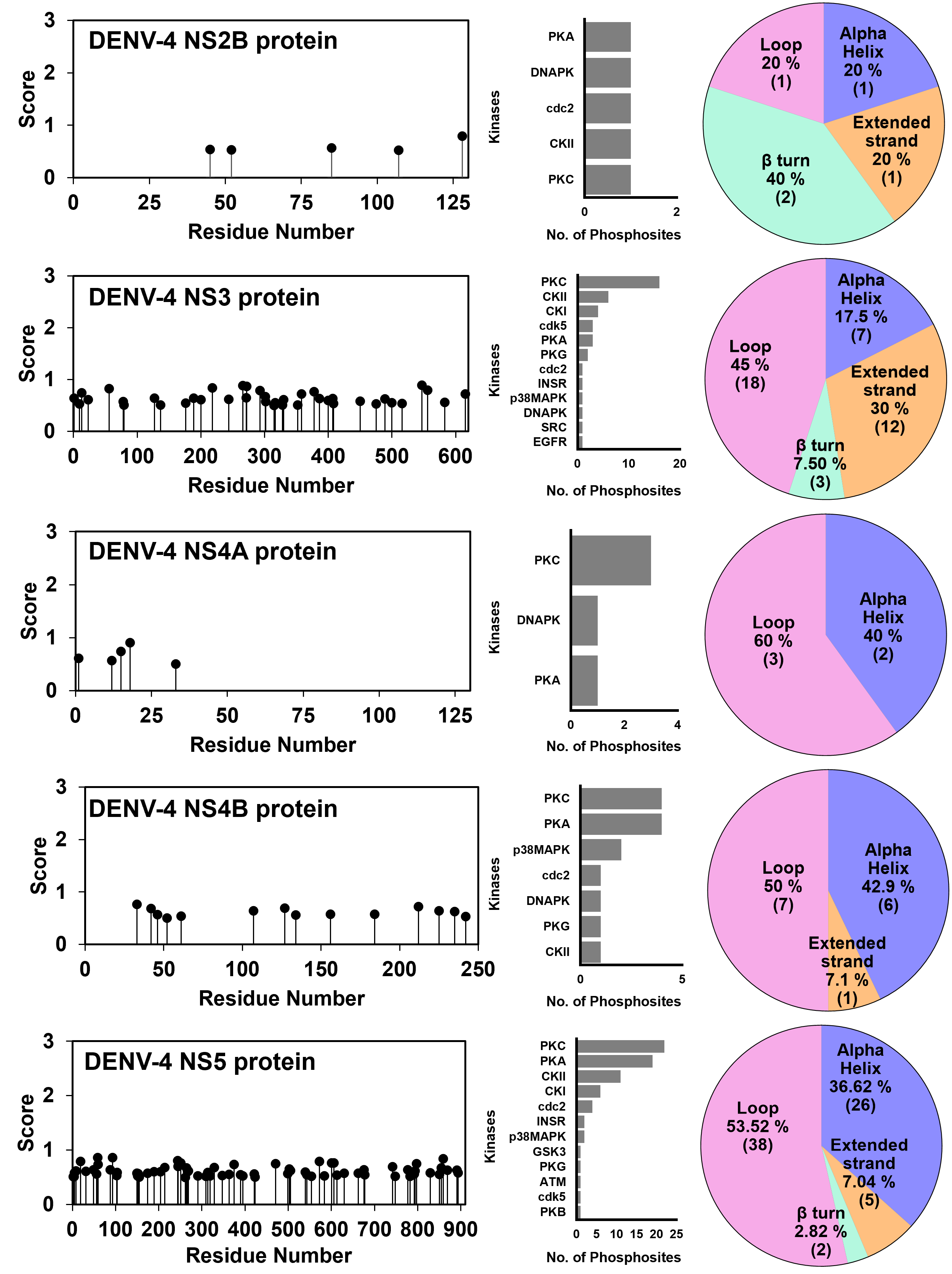

Supplement: S8 Fig — Left panel – Lollipop graphs showing the phosphosites predicted using NetPhos3.1; Middle panel – The best hit kinases for each of the predicted phosphosites and, Right panel – the distribution of secondary structural elements in the viral proteins (pink-loop; cyan-beta turns; orange-extended strand; blue- alpha helix) analyzed using SOPMA secondary structure prediction software. Also indicated are the total number of phosphosites that are present in a particular secondary structure. (TIF) [file pone.0345872.s008.tif]

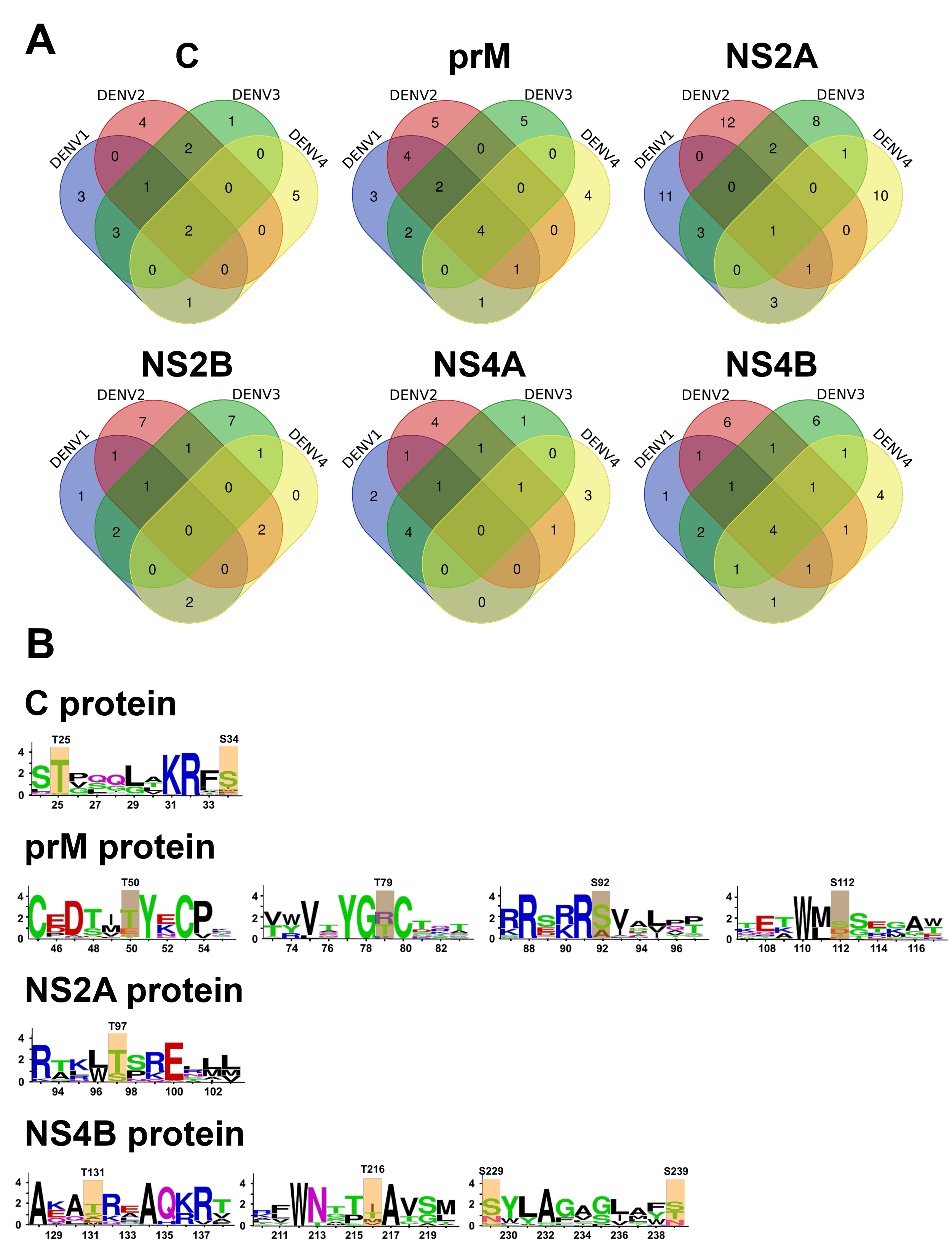

Supplement: S9 Fig — (A) Venn diagram showing the predicted phosphosites for the C, prM, NS2A, NS2B, NS4A, and NS4B in Dengue virus serotypes 1, 2, 3, and 4. Note the number of overlapping phosphosites across multiple DENV serotypes. (B) Web logo showing the phosphorylation cluster across the sequences in the C, prM, NS2A, and NS4B proteins of flaviviruses. The sequence motifs near the conserved phosphosites in DENV serotypes are shown. The conserved phosphosites from the DENV serotypes are highlighted in an orange box, and the bits show the degree of conservation across flaviviruses (a bit value of 4 represents a highly conserved residue, whereas a value of 1 shows the least conservation at that position). (TIF) [file pone.0345872.s009.tif]

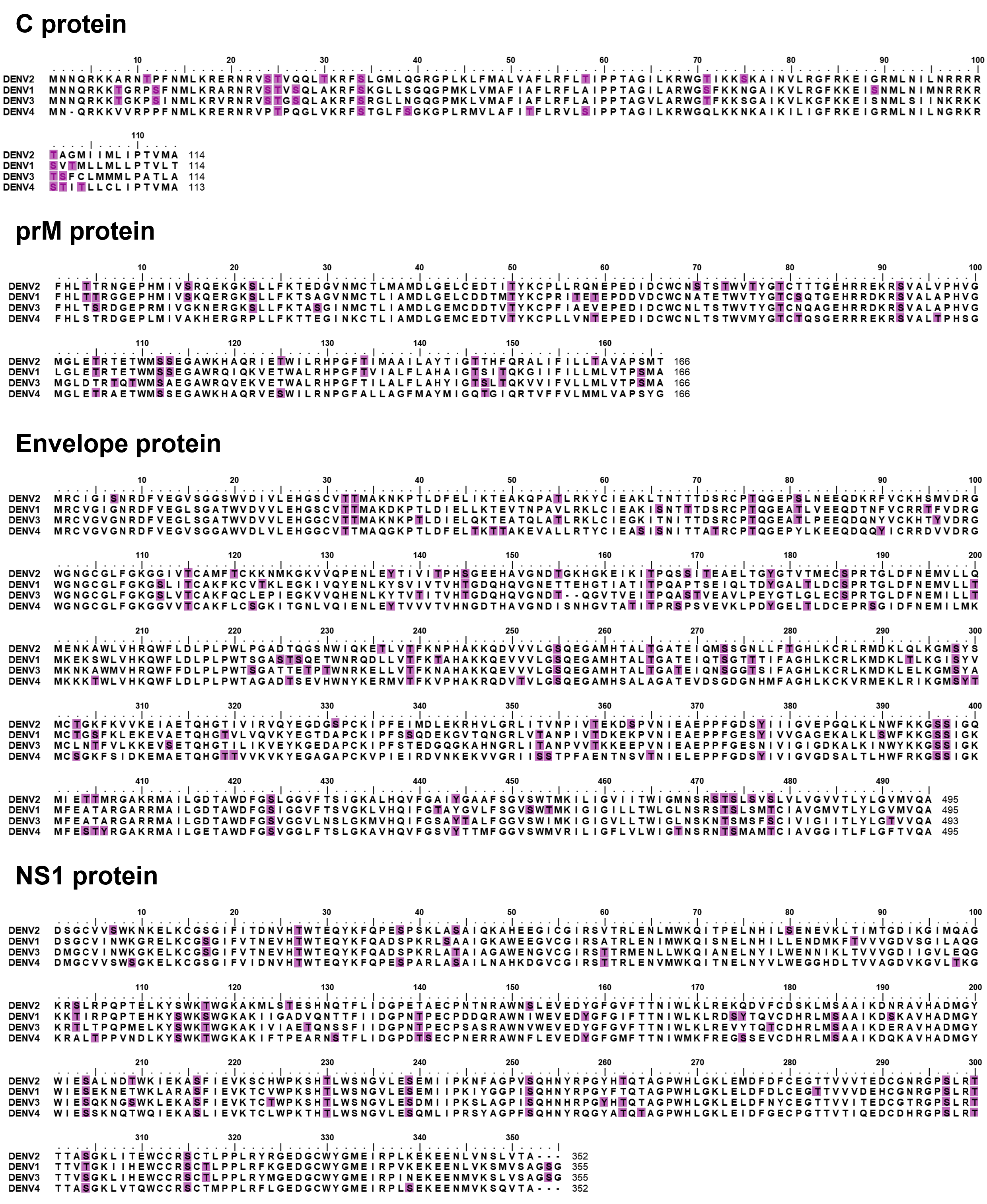

Supplement: S10 Fig — Multiple sequence alignment of Dengue viral proteins (serotypes 1, 2, 3, and 4) Capsid, prM, glycoprotein E, NS1. (TIF) [file pone.0345872.s010.tif]

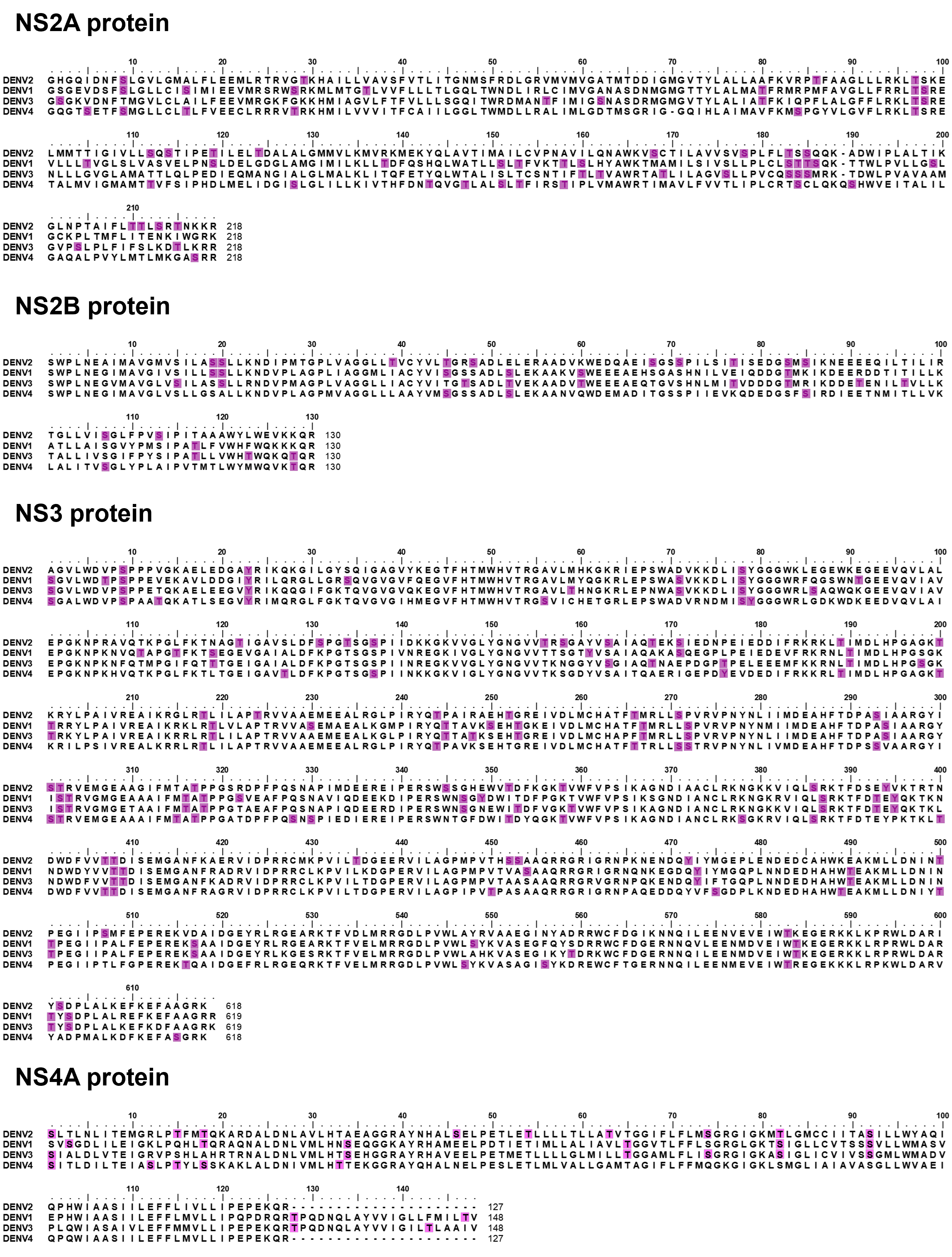

Supplement: S11 Fig — Multiple sequence alignment of Dengue viral proteins (serotypes 1, 2, 3, and 4) NS2A, NS2B, NS3, and NS4A. (TIF) [file pone.0345872.s011.tif]

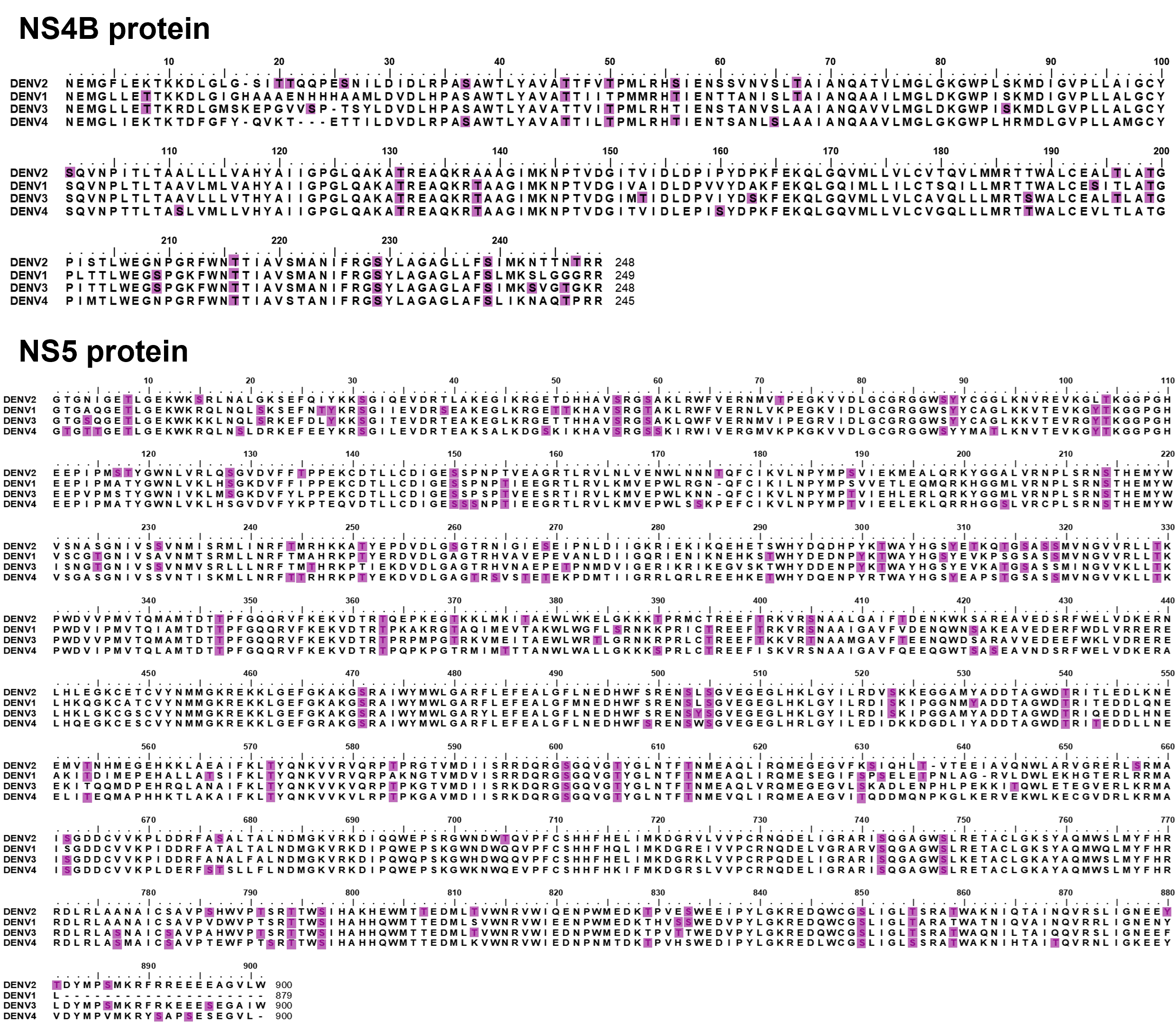

Supplement: S12 Fig — Multiple sequence alignment of Dengue viral proteins (serotypes 1, 2, 3, and 4) NS4B, and NS5. (TIF) [file pone.0345872.s012.tif]

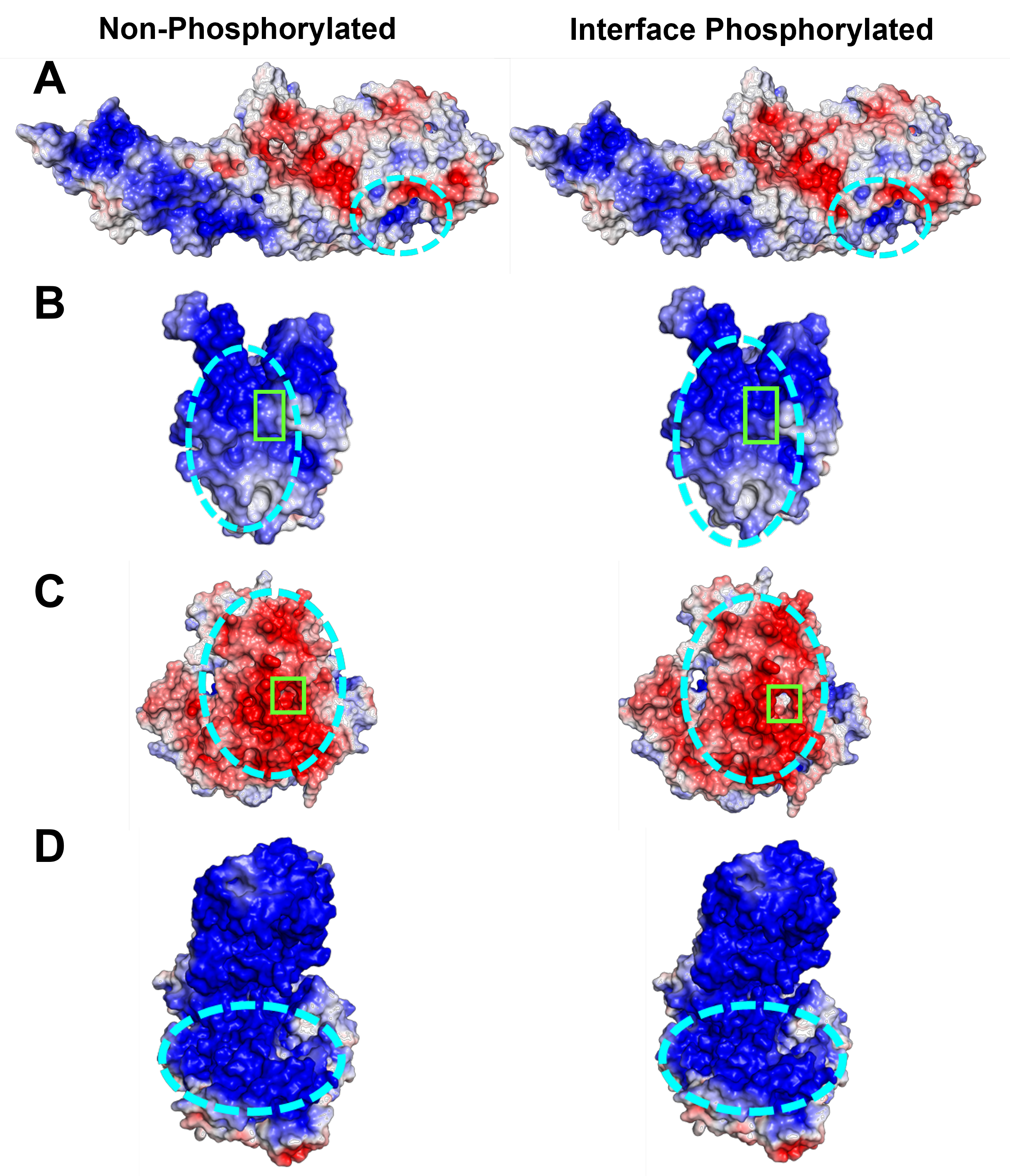

Supplement: S13 Fig — Surface electrostatic potential of Dengue viral proteins (A) glycoprotein E (PDB ID: 7CTH), (B) NS3 protease (PDB ID: 2FOM), (C) NS3 helicase (PDB ID: 8GZQ), and (D) NS5 (PDB ID: 8T12) in non-phosphorylated (left panel) and interface phosphorylated (right panel) states. The interaction interface is highlighted in a cyan circle, and the changes in electrostatic potential are highlighted in the green box. Blue represents positive charge potential, and red represents negative charge potential. The inputs for computing the electrostatic potential using the Advanced Poisson-Boltzmann Solver (APBS) were generated with the PDB2PQR tool. (TIF) [file pone.0345872.s013.tif]

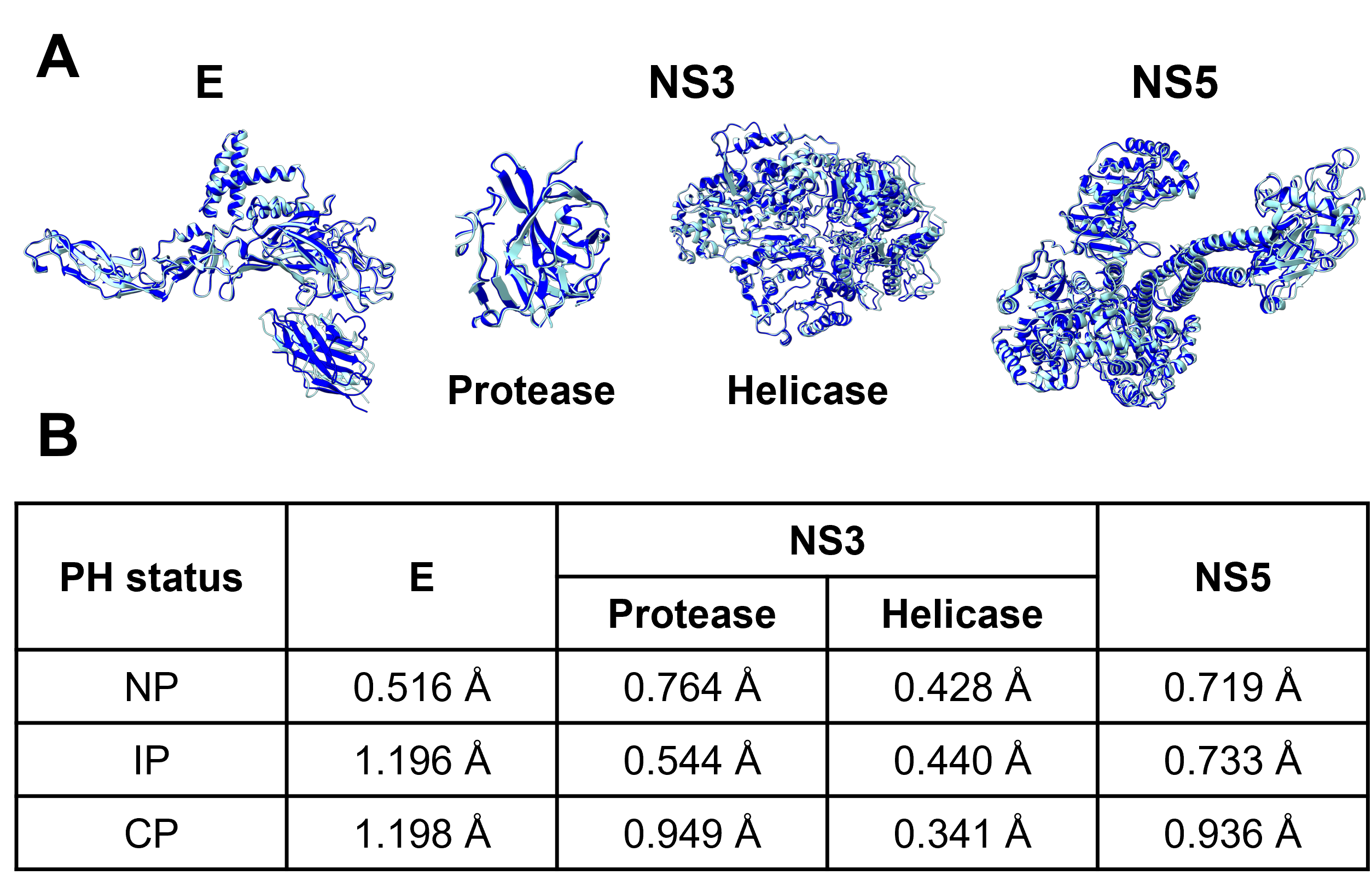

Supplement: S14 Fig — (A) Overlay of non-phosphorylated crystal structure (cyan) and HADDOCK docked (blue) of glycoprotein E: mAb ScFv EDE1 C10 (PDB ID: 7CTH), NS3 protease: NS2B (PDB ID: 2FOM), NS3 helicase: NS5 (PDB ID: 8GZQ) and NS5:hSTAT2 (PDB ID: 8T12). Note that all the structures overlay well suggesting that HADDOCK generated models are similar to that of the crystal structures. (B) The Cα backbone RMSD of the overlay of PDB structures with interface/completely phosphorylated DENV proteins shows that the structures generated for phosphorylated states are very similar to the non-phosphorylated DENV proteins. (TIF) [file pone.0345872.s014.tif]

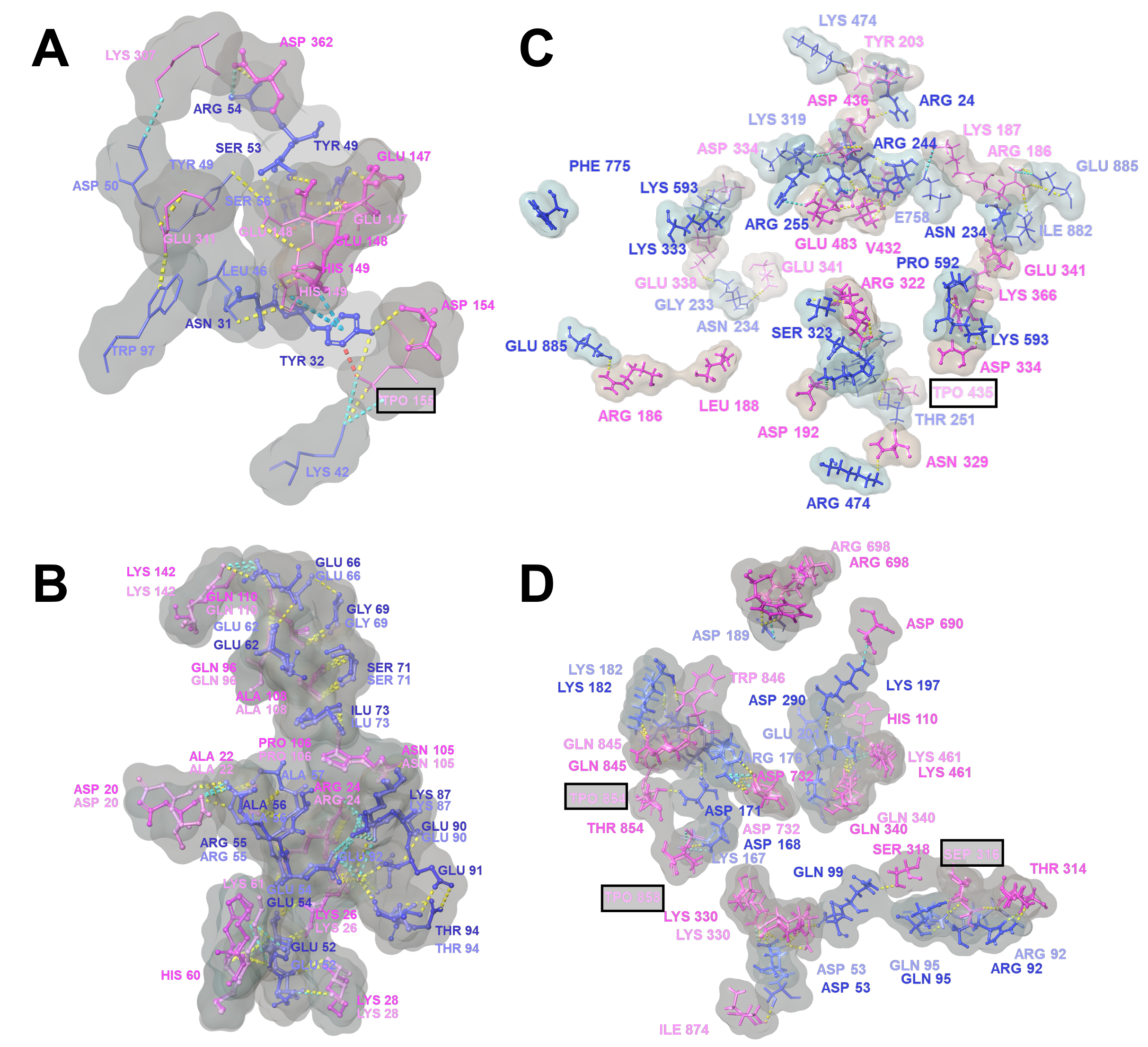

Supplement: S15 Fig — Surface and stick representation of interface in (A) glycoprotein E in complex with the scFv fragment of the mAb EDE1 C10, (B) NS3 protease in complex with NS2B, (C) NS3 helicase in complex with NS5, (D) NS5 in complex with human STAT2. The non-phosphorylated residues of dengue proteins are shown in the thin pink ball and stick model, whereas the phosphorylated residues are shown in the thick light pink ball and stick model. The residues of the interacting proteins of non-phosphorylated protein are shown in a thin royal blue ball and stick model, whereas the residues associated with phosphorylated proteins are shown in thick sky-blue ball and stick model. Also shown is the surface of Dengue proteins (light golden – phosphorylated and dark golden – non-phosphorylated) and interacting proteins (light cyan – phosphorylated and dark cyan – non-phosphorylated). The interacting residues are also labeled. Note that the NS3 protease lacks phosphorylation sites at the interface but shows differences in the interaction due to the phosphorylation of other residues due to long-range allosteric effects. (TIF) [file pone.0345872.s015.tif]

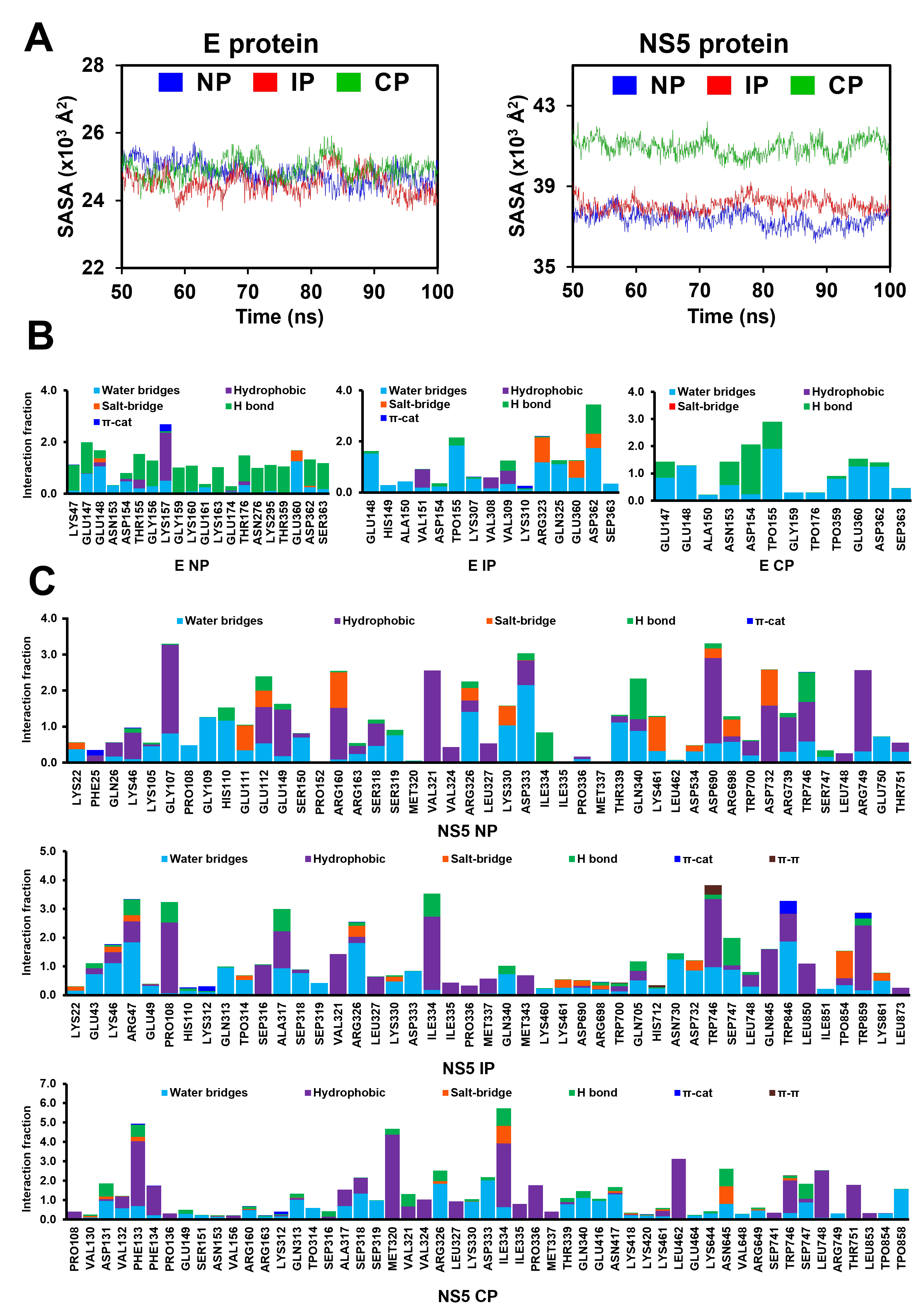

Supplement: S16 Fig — (A) Solvent accessible surface area (SASA) plot for the glycoprotein E and NS5 in the glycoprotein E: mAb ScFv EDE1 C10 (left panel) and NS5:hSTAT2 (right panel) for the last 50 ns of the MD run. The complexes in the non-phosphorylated (blue), interface phosphorylated (red), and completely phosphorylated (green) states of the glycoprotein E and NS5 showed less variability of the SASA during the course of the MD run. Plot showing the residues that are involved in various types of interactions like the π-cation interaction (blue), hydrogen bond (purple), salt bridges (orange), water bridges (cyan), and hydrophobic (green) interactions for the (B) glycoprotein E: mAb ScFv EDE1 C10 (left panel) and (C) NS5:hSTAT2. Note that both these complexes have higher interaction fractions for the interaction of residues with the water molecules. (TIF) [file pone.0345872.s016.tif]

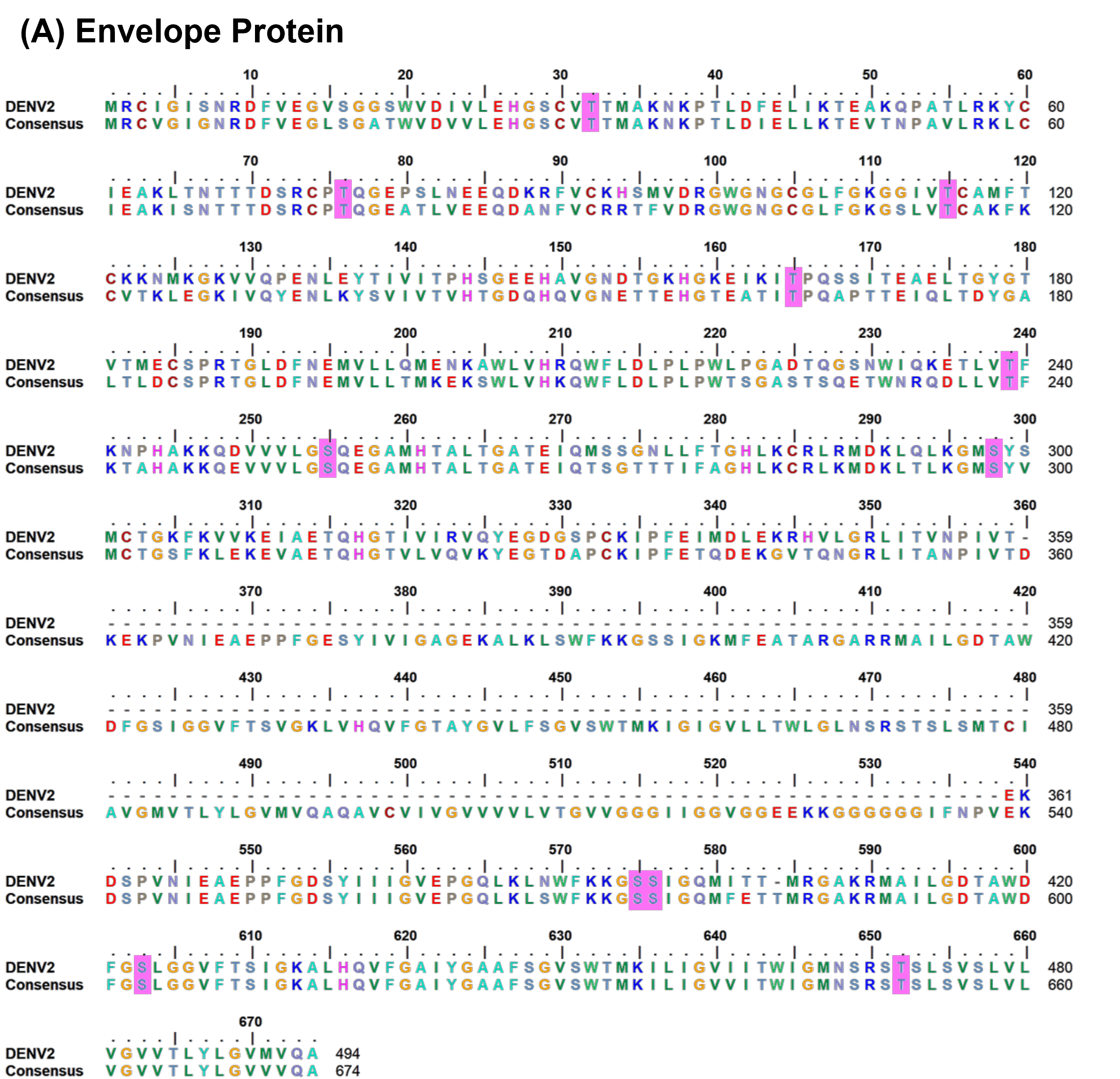

Supplement: S17 Fig — Sequence alignment of consensus sequence of circulating strains of DENV serotypes with DENV2 sequence for Envelope glycoprotein E. The conserved phosphosites in DENV-2 are highlighted in magenta box. Note that the predicted phosphosites are highly conserved across the circulating strains of DENV serotypes. (TIF) [file pone.0345872.s017.tif]

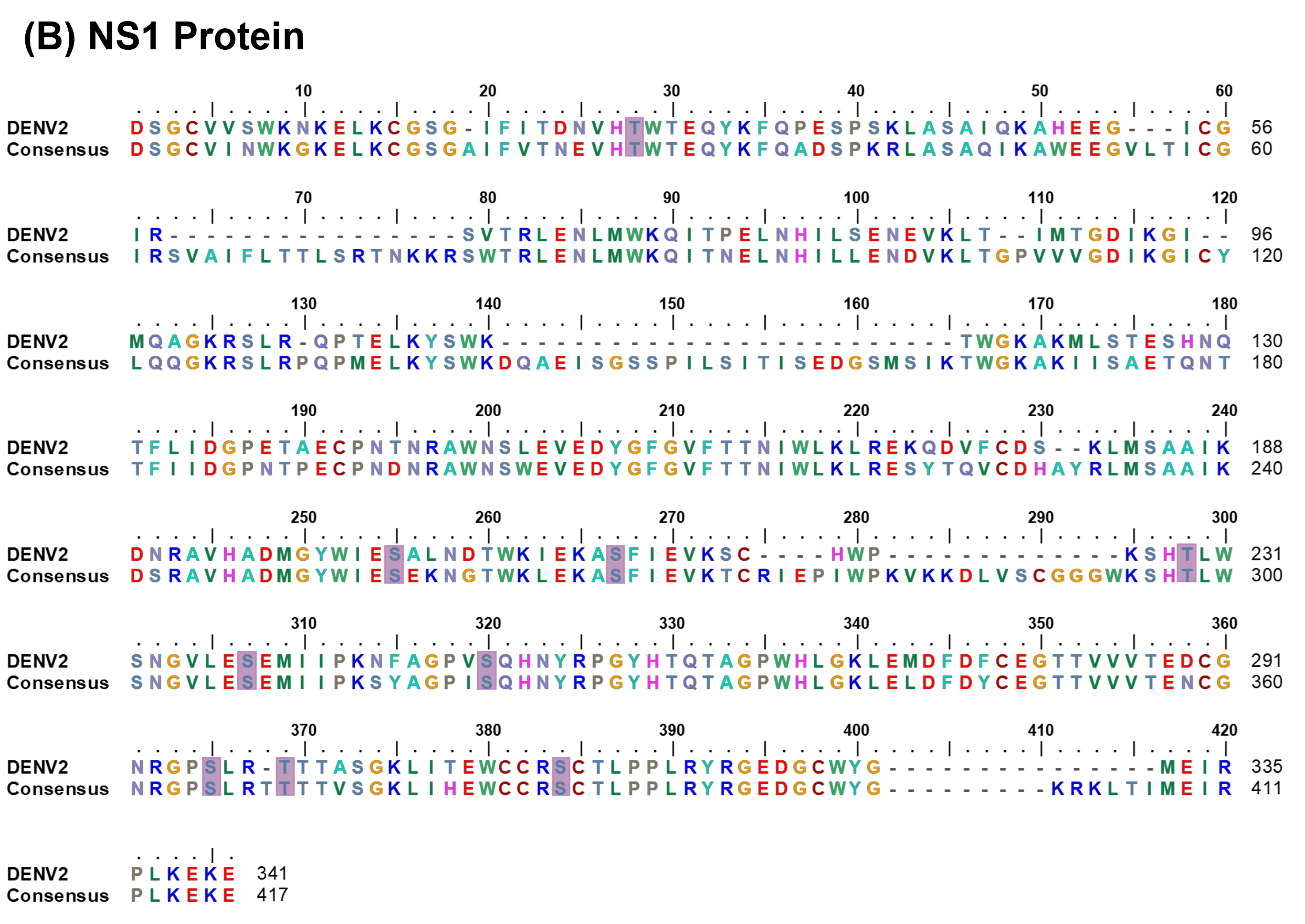

Supplement: S18 Fig — Sequence alignment of consensus sequence of circulating strains of DENV serotypes with DENV2 sequence for NS1.The conserved phosphosites in DENV-2 are highlighted in magenta box. Note that the predicted phosphosites are highly conserved across the circulating strains of DENV serotypes. (TIF) [file pone.0345872.s018.tif]

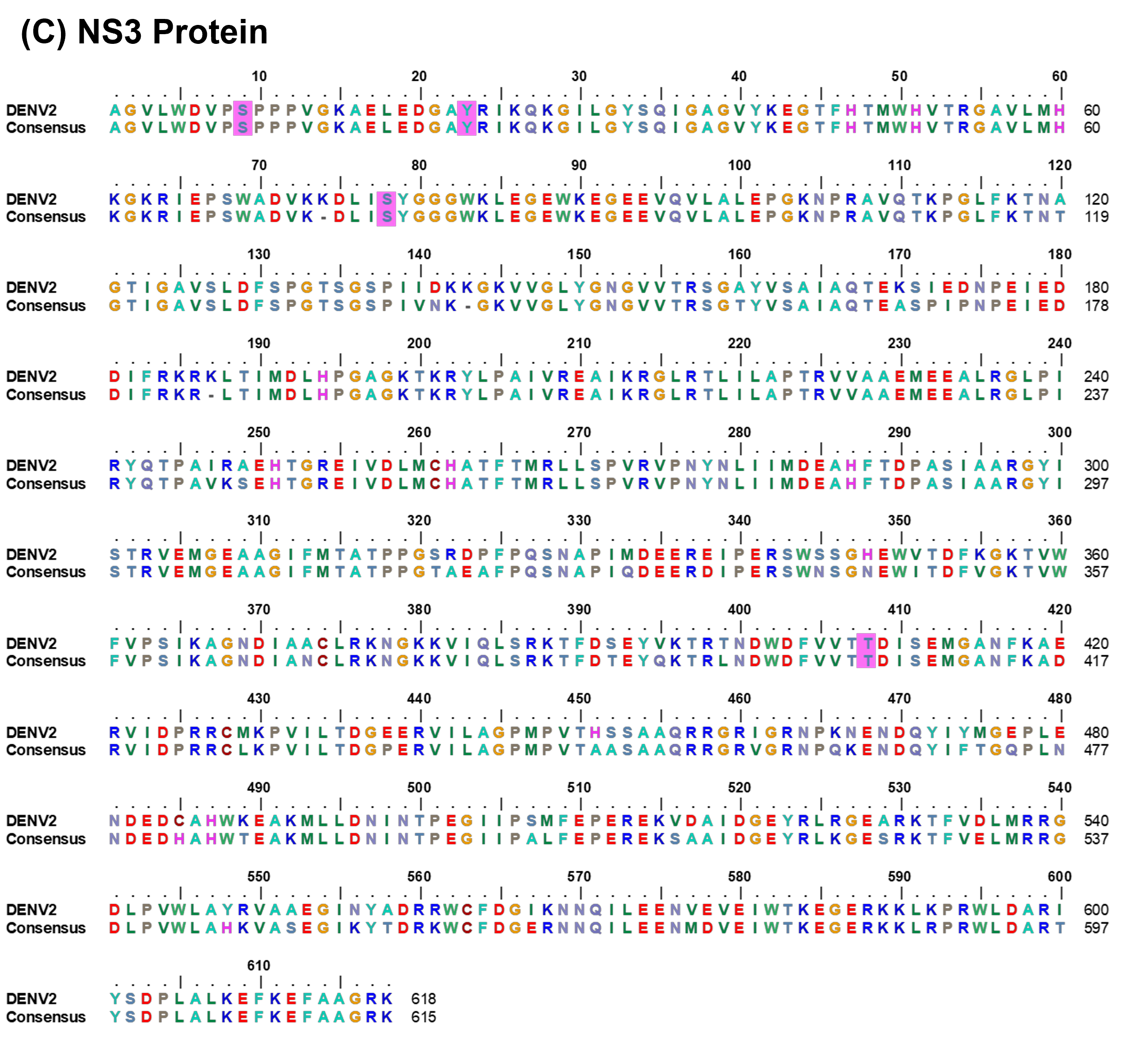

Supplement: S19 Fig — Sequence alignment of consensus sequence of circulating strains of DENV serotypes with DENV2 sequence for NS3. The conserved phosphosites in DENV-2 are highlighted in magenta box. Note that the predicted phosphosites are highly conserved across the circulating strains of DENV serotypes. (TIF) [file pone.0345872.s019.tif]

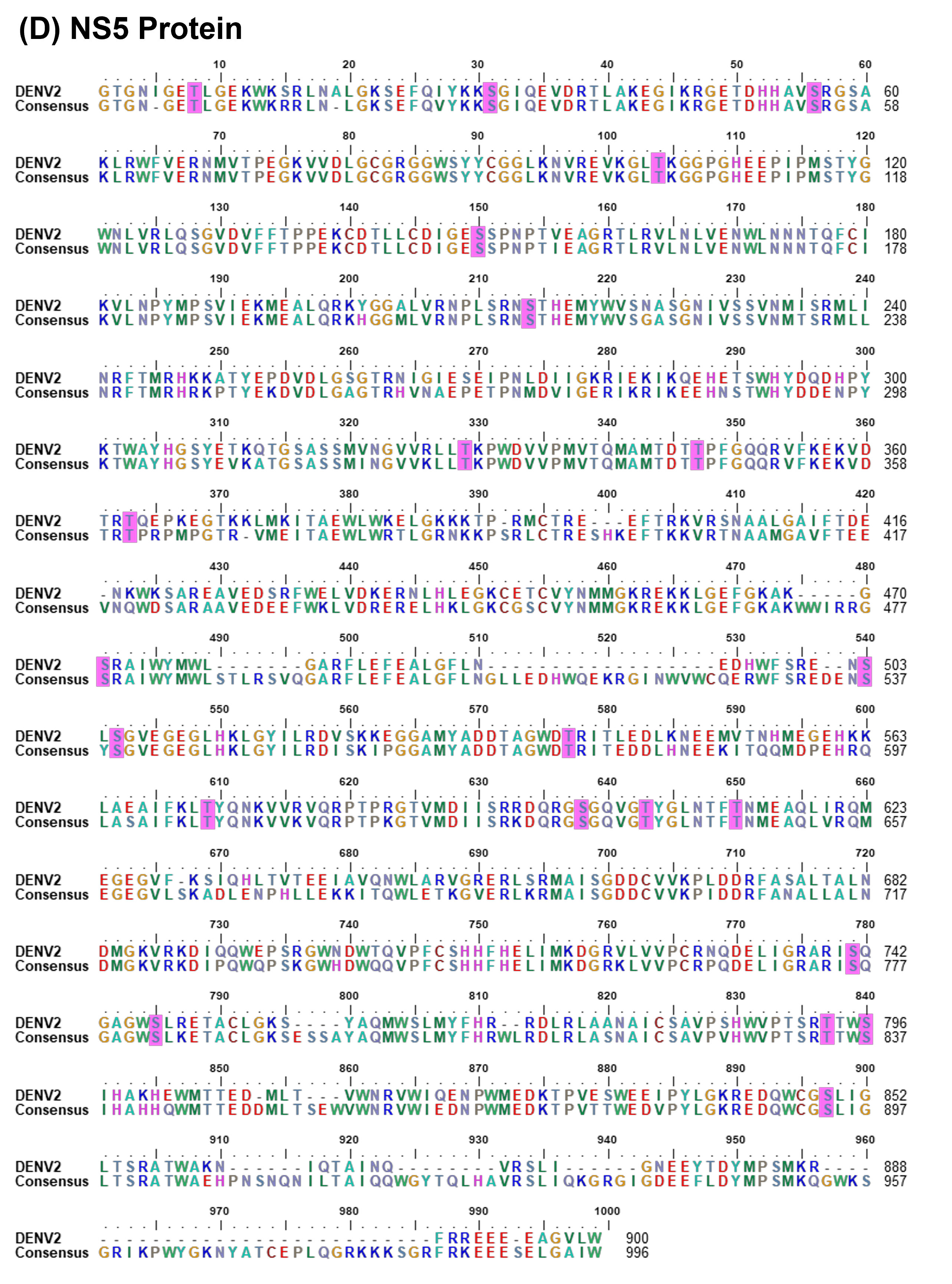

Supplement: S20 Fig — Sequence alignment of consensus sequence of circulating strains of DENV serotypes with DENV2 sequence for NS5. The conserved phosphosites in DENV-2 are highlighted in magenta box. Note that the predicted phosphosites are highly conserved across the circulating strains of DENV serotypes. (TIF) [file pone.0345872.s020.tif]
