# Supplementary material for: Phosphorylation landscape of dengue virus proteins and their implications in protein-protein interactions
Source: PLoS One. 2026 May 12;21(5):e0345872. doi: 10.1371/journal.pone.0345872 (PMC13166905; doi:10.1371/journal.pone.0345872)
Supplement: S1 Table — (DOCX) [file pone.0345872.s021.docx]

**S1 Table: Distribution of phosphosites across the various viral proteins of DENV-1.**

| **DENV Protein** | **No. of Amino Acids (y)** | **No. of Potential Phosphorylation Sites (x)** | **(x)/(y) (%)** | **Residues Phosphorylated** |
| --- | --- | --- | --- | --- |
| C Protein | 114 | 10 | 8.77 | T8, S12, S24, T25, S27, S34, S71, S89, S101, T103 |
| prM Protein | 166 | 17 | 10.24 | T4, T5, S15, S22, T50, T57, T59, T79, S81, S92, T105, S112, S113, T134, T146, T149, S164 |
| E Protein | 495 | 44 | 8.89 | T32, T33, S66, T69, T76, T81, T95, S112, T115, T123, T145, T165, Y178, T182, S186, T200, S225, T226, S227, T239, T242, S255, T265, S273, T276, T293, S298, T303, S305, T319, S339, T353, T359, Y377, S390, S396, S397, S424, T442, S452, T454, T473, S474, T478 |
| NS1 | 355 | 25 | 7.04 | S17, T27, S43, T87, T103, S114, S117, T140, Y158, S174, Y175, S185, S191, S204, S216, T230, S239, S252, T283, S297, T300, T304, S315, T317, S354 |
| NS2A | 218 | 19 | 8.72 | S9, S16, S28, T36, T80, T97, S98, T105, S119, T138, S151, T153, T158, S160, S183, T184, T185, S186, S198 |
| NS2B | 130 | 7 | 5.38 | S19, S20, S45, S52, S60, T83, T117 |
| NS3 | 619 | 44 | 7.11 | S1, T7, S9, Y23, S34, S71, S78, T91, T111, T115, S119, Y161, S171, T190, T201, T219, S230, T245, S250, T253, T267, S272, S294, S302, T303, T316, T318, S322, S347, Y349, S387, T393, Y395, T408, T409, S454, Y473, T490, T501, S517, S548, T584, T601, S603 |
| NS4A | 148 | 8 | 5.41 | S3, T18, S34, T65, S82, S92, T128, T147 |
| NS4B | 249 | 12 | 4.81 | T8, T46, T56, T67, T131, T138, S194, T199, S209, T216, S229, S239 |
| NS5 | 879 | 56 | 6.37 | T8, S21, T27, Y28, S31, S39, T50, T51, S56, T59, Y89, Y103, T104, S128, S150, T155, S213, T224, T250, T290, Y299, T301, S307, S318, T328, T346, T362, T369, S385, T394, T399, S404, S420, S470, S502, S504, S522, Y530, T539, T553, T565, T571, S600, T605, T612, S629, S631, T635, S740, S746, T792, S795, S830, S831, S848, T853 |
