# Supplementary material for: Phosphorylation landscape of dengue virus proteins and their implications in protein-protein interactions
Source: PLoS One. 2026 May 12;21(5):e0345872. doi: 10.1371/journal.pone.0345872 (PMC13166905; doi:10.1371/journal.pone.0345872)
Supplement: S2 Table — (DOCX) [file pone.0345872.s022.docx]

**S2 Table: Distribution of phosphosites across the various viral proteins of DENV-3.**

| **DENV Protein** | **No. of Amino Acids (y)** | **No. of Potential Phosphorylation Sites (x)** | **(x)/(y) (%)** | **Residues Phosphorylated** |
| --- | --- | --- | --- | --- |
| C Protein | 114 | 9 | 7.89 | T8, S12, S24, T25, S27, S34, T71, T101, S102 |
| prM Protein | 166 | 13 | 7.83 | S5, S22, S29, T50, T79, S92, T107, T109, S112, T146, S147, T149, S164 |
| E Protein | 493 | 39 | 7.91 | T32, T33, T40, T55, T76, T81, Y96, S112, T115, T140, T145, T155, T163, S167, T168, S184, T198, S220, T226, T228, T237, S253, T263, T266, S271, T274, S296, T303, S311, T351, T357, S394, S395, S422, Y442, T443, T471, S476, T489 |
| NS1 | 355 | 26 | 7.32 | S17, T27, T44, T60, T103, S114, T117, T128, T140, T178, S185, S204, S209, S216, T224, T230, S239, S252, Y260, T262, S297, T300, S304, S315, T317, S354 |
| NS2A | 218 | 15 | 6.88 | S2, T56, S62, T80, T97, S98, T160, T162, T169, S176, S183, S184, S185, S203, T214 |
| NS2B | 130 | 12 | 9.23 | S15, S20, T47, T52, T60, T77, T83, T91, T96, T117, T123, T128 |
| NS3 | 619 | 41 | 6.62 | S1, S9, Y23, T59, S71, S78, S86, T119, S163, T168, T176, T190, S198, T201, T219, T245, T248, T253, T267, S272, S294, S302, T303, T316, T318, S347, T353, T359, S387, T393, Y395, T408, T409, Y473, T490, T501, S517, T559, T584, T601, S603 |
| NS4A | 148 | 8 | 5.41 | S1, S34, T65, S74, S82, S92, T128, T143 |
| NS4B | 248 | 17 | 6.85 | T8, S23, T49, S85, T130, T137, T152, S162, S187, T195, T198, S208, T215, S228, S238, S242, T245 |
| NS5 | 900 | 60 | 6.67 | S4, T8, S21, Y28, S31, S56, S59, Y89, Y103, T104, S128, S150, T155, T188, S213, T224, S230, T245, T270, Y299, T301, T313, S315, T328, T346, T362, T369, T383, T394, T399, T404, T413, S470, S502, Y503, S504, S522, T539, T571, T583, S600, T605, T612, S629, T644, S661, S741, S747, S776, S781, T790, T793, S796, T811, T831, S849, T854, T858, S885, S895 |
