# Supplementary material for: Phosphorylation landscape of dengue virus proteins and their implications in protein-protein interactions
Source: PLoS One. 2026 May 12;21(5):e0345872. doi: 10.1371/journal.pone.0345872 (PMC13166905; doi:10.1371/journal.pone.0345872)
Supplement: S3 Table — (DOCX) [file pone.0345872.s023.docx]

**S3 Table: Distribution of phosphosites across the various viral proteins of DENV-4**

| **DENV Protein** | **No. of Amino Acids (y)** | **No. of Potential Phosphorylation Sites (x)** | **(x)/(y) (%)** | **Residues Phosphorylated** |
| --- | --- | --- | --- | --- |
| C Protein | 113 | 8 | 7.08 | T24, S33, S38, T51, S57, S100, T101, T103 |
| prM Protein | 166 | 10 | 6.02 | T50, T59, T79, T81, S92, T96, T105, S112, S125, T147 |
| E Protein | 495 | 44 | 8.89 | T32, T46, T48, T49, S64, S66, T72, T76, Y90, T115, S122, Y137, T163, T165, S168, Y178, T182, S189, T205, T226, T239, T251, S255, S298, Y299, T300, S303, T319, T320, S353, S354, T365, Y377, S396, S397, S404, T405, Y406, S424, Y444, T468, T473, S474, T478 |
| NS1 | 352 | 27 | 7.67 | S9, T27, S38, S44, T60, T98, T105, S114, T117, S131, T140, S141, Y158, S175, S185, S204, S216, T230, S239, S252, T262, T264, S297, T300, S304, S315, S339 |
| NS2A | 218 | 16 | 7.34 | S5, S9, T16, T28, S83, T96, T111, S127, T142, T146, S150, T152, T157, S183, S189, S216 |
| NS2B | 130 | 5 | 3.85 | S45, S52, S85, S107, T128 |
| NS3 | 618 | 40 | 6.47 | S1, S9, T13, Y23, S56, S78, Y79, T127, S137, Y176, T189, T200, T218, T244, T266, S271, S272, S293, S301, T302, T315, T317, S328, S330, T352, T358, S378, S386, T400, T407, T408, T450, S475, T489, T500, T516, S547, S556, T583, S615 |
| NS4A | 127 | 5 | 3.94 | S1, S12, T15, S18, T33 |
| NS4B | 245 | 14 | 5.71 | S33, T42, T46, T52, S61, S107, T127, T134, S156, T184, T212, S225, S235, T242 |
| NS5 | 900 | 71 | 7.89 | T2, T4, T5, T8, S19, S31, S49, S56, S59, S60, S88, T93, Y103, T104, S150, S151, S152, T155, S174, T189, S204, S214, T244, T245, T251, T262, S264, T267, T269, T291, Y309, T314, S319, T329, T347, T363, T375, S390, T395, S421, S423, S471, S499, S503, S505, T540, T543, T554, T572, T584, S601, T606, T613, T630, S662, S676, T677, S742, S748, S777, S782, S792, T794, S797, T829, S850, S855, T859, T869, S891, S894 |
