# Supplementary material for: Phosphorylation landscape of dengue virus proteins and their implications in protein-protein interactions
Source: PLoS One. 2026 May 12;21(5):e0345872. doi: 10.1371/journal.pone.0345872 (PMC13166905; doi:10.1371/journal.pone.0345872)
Supplement: S8 Table — (DOCX) [file pone.0345872.s028.docx]

**S8 Table: Accession IDs for the NS2A protein sequences used in the phosphosite evolutionary conservation analysis.**

| **NCBI accession id** | **Flavivirus strain information** |
| --- | --- |
| P14340.2 | Dengue virus 2 Thailand/NGS-C/1944 |
| P12823.1 | Dengue virus 2 Puerto Rico/PR159-S1/1969 |
| P27914.1 | Dengue virus 2 Tonga/EKB194/1974 |
| P14337.2 | Dengue virus 2 Thailand/0168/1979 |
| P07564.2 | Dengue virus 2 Jamaica/1409/1983 |
| P29991.1 | Dengue virus 2 16681-PDK53 |
| Q9WDA6.1 | Dengue virus 2 Peru/IQT2913/1996 |
| P29990.1 | Dengue virus 2 Thailand/16681/84 |
| Q6YMS3.1 | Dengue virus 3 Martinique/1243/1999 |
| Q6YMS4.1 | Dengue virus 3 Sri Lanka/1266/2000 |
| Q99D35.1 | Dengue virus 3 China/80-2/1980 |
| P27915.1 | Dengue virus 3 Philippines/H87/1956 |
| Q5UB51.1 | Dengue virus 3 Singapore/8120/1995 |
| P27909.2 | Dengue virus 1 Brazil/97-11/1997 |
| P33478.2 | Dengue virus 1 Singapore/S275/1990 |
| P17763.2 | Dengue virus 1 Nauru/West Pac/1974 |
| Q2YHF2.1 | Dengue virus 4 Thailand/0476/1997 |
| Q5UCB8.1 | Dengue virus 4 Singapore/8976/1995 |
| P09866.2 | Dengue virus 4 Dominica/814669/1981 |
| Q2YHF0.1 | Dengue virus 4 Thailand/0348/1991 |
| Q58HT7.1 | Dengue virus 4 Philippines/H241/1956 |
| Q32ZD5.1 | Kokobera virus |
| P09732.2 | St. Louis encephalitis virus (strain MS1-7) |
| Q32ZE1.1 | Zika virus |
| A0A024B7W1.1 | Zika virus ZIKV/H. sapiens/FrenchPolynesia/10087PF/2013 |
| A0A142I5B9.1 | Zika virus ZIKV/Human/Cambodia/FSS13025/2010 |
| Q9Q6P4.2 | West Nile virus strain NY-99 |
| P06935.2 | West Nile virus |
| P14335.1 | Kunjin virus (STRAIN MRM61C) |
| P03314.1 | Yellow fever virus 17D |
| Q6DV88.1 | Yellow fever virus strain Ghana/Asibi/1927 |
| Q6J3P1.1 | Yellow fever virus isolate Ivory Coast/1999 |
| Q89277.2 | Yellow fever virus strain French neurotropic vaccine |
| Q9YRV3.1 | Yellow fever virus Trinidad/79A/1979 |
| Q98803.1 | Yellow fever virus isolate Ivory Coast/85-82H/1982 |
| Q1X881.1 | Yellow fever virus isolate Angola/14FA/1971 |
| Q074N0.1 | Yellow fever virus isolate Ethiopia/Couma/1961 |
| Q1X880.1 | Yellow fever virus isolate Uganda/A7094A4/1948 |
