# Supplementary material for: Phosphorylation landscape of dengue virus proteins and their implications in protein-protein interactions
Source: PLoS One. 2026 May 12;21(5):e0345872. doi: 10.1371/journal.pone.0345872 (PMC13166905; doi:10.1371/journal.pone.0345872)
Supplement: S11 Table — (DOCX) [file pone.0345872.s031.docx]

**S11 Table: Accession IDs for the NS5 protein sequences used in the phosphosite evolutionary conservation analysis.**

| **NCBI accession id** | **Flavivirus strain information** |
| --- | --- |
| P14340.2 | Dengue virus 2 Thailand/NGS-C/1944 |
| P14337.2 | Dengue virus 2 Thailand/0168/1979 |
| P07564.2 | Dengue virus 2 Jamaica/1409/1983 |
| P29991.1 | Dengue virus 2 16681-PDK53 |
| P29990.1 | Dengue virus 2 Thailand/16681/84 |
| P12823.1 | Dengue virus 2 Puerto Rico/PR159-S1/1969 |
| Q9WDA6.1 | Dengue virus 2 Peru/IQT2913/1996 |
| P27915.1 | Dengue virus 3 Philippines/H87/1956 |
| Q99D35.1 | Dengue virus 3 China/80-2/1980 |
| Q6YMS4.1 | Dengue virus 3 Sri Lanka/1266/2000 |
| Q5UB51.1 | Dengue virus 3 Singapore/8120/1995 |
| Q6YMS3.1 | Dengue virus 3 Martinique/1243/1999 |
| P27909.2 | Dengue virus 1 Brazil/97-11/1997 |
| P17763.2 | Dengue virus 1 Nauru/West Pac/1974 |
| P33478.2 | Dengue virus 1 Singapore/S275/1990 |
| Q5UCB8.1 | Dengue virus 4 Singapore/8976/1995 |
| P09866.2 | Dengue virus 4 Dominica/814669/1981 |
| Q58HT7.1 | Dengue virus 4 Philippines/H241/1956 |
| Q2YHF2.1 | Dengue virus 4 Thailand/0476/1997 |
| Q2YHF0.1 | Dengue virus 4 Thailand/0348/1991 |
| Q32ZD4.1 | Rocio virus |
| Q32ZD7.1 | Ilheus virus |
| P14335.1 | Kunjin virus (STRAIN MRM61C) |
| Q9Q6P4.2 | West Nile virus strain NY-99 |
| P09732.2 | St. Louis encephalitis virus (strain MS1-7) |
| Q5WPU5.1 | Usutu virus |
| P06935.2 | West Nile virus |
| P05769.2 | Murray valley encephalitis virus (strain MVE-1-51) |
| Q32ZE0.1 | Bussuquara virus |
| P27395.1 | Japanese encephalitis virus strain SA-14 |
| P19110.1 | Japanese encephalitis virus strain SA(V) |
| G3FEX6.1 | Japanese encephalitis virus strain M28 |
| P32886.1 | Japanese encephalitis virus strain JAOARS982 |
| G1CRV4.1 | Tembusu virus |
| A0A024B7W1.1 | Zika virus ZIKV/H. sapiens/FrenchPolynesia/10087PF/2013 |
| A0A142I5B9.1 | Zika virus ZIKV/Human/Cambodia/FSS13025/2010 |
| Q32ZE1.1 | Zika virus |
| Q32ZD5.1 | Kokobera virus |
| C5H431.1 | Wesselsbron virus |
| Q1X881.1 | Yellow fever virus isolate Angola/14FA/1971 |
| Q1X880.1 | Yellow fever virus isolate Uganda/A7094A4/1948 |
| Q074N0.1 | Yellow fever virus isolate Ethiopia/Couma/1961 |
| Q89277.2 | Yellow fever virus strain French neurotropic vaccine |
| P03314.1 | Yellow fever virus 17D |
| Q98803.1 | Yellow fever virus isolate Ivory Coast/85-82H/1982 |
| Q9YRV3.1 | Yellow fever virus Trinidad/79A/1979 |
| Q6DV88.1 | Yellow fever virus strain Ghana/Asibi/1927 |
| Q6J3P1.1 | Yellow fever virus isolate Ivory Coast/1999 |
| C8XPB2.1 | Edge Hill virus |
| C8XPA8.1 | Banzi virus |
| P29837.2 | Langat virus (strain TP21) |
| Q04538.1 | Tick-borne powassan virus (strain lb) |
| D7RF80.1 | Kyasanur Forest disease virus |
| Q91B85.1 | Alkhumra hemorrhagic fever virus |
| Q7T6D2.1 | Omsk hemorrhagic fever virus |
| P14336.4 | Tick-borne encephalitis virus (WESTERN SUBTYPE) |
| Q01299.1 | Tick-borne encephalitis virus (strain HYPR) |
| P07720.3 | Tick-borne encephalitis virus (STRAIN SOFJIN) |
| P22338.2 | Louping ill virus |
| P33515.1 | Cell fusing agent virus |
